# Why are cell populations maintained via multiple compartments?

**DOI:** 10.1098/rsif.2022.0629

**Published:** 2022-11-09

**Authors:** Flavia Feliciangeli, Hanan Dreiwi, Martín López-García, Mario Castro Ponce, Carmen Molina-París, Grant Lythe

**Affiliations:** ^1^ School of Mathematics, University of Leeds, Leeds LS2 9JT, UK; ^2^ Systems Pharmacology and Medicine, Bayer AG, Leverkusen 51368, Germany; ^3^ Instituto de Investigación Tecnológica (ITT), Universidad Pontificia Comillas, Madrid, Spain; ^4^ T-6, Theoretical Biology and Biophysics, Los Alamos National Laboratory, Los Alamos, NM 87545, USA

**Keywords:** branching process, probability generating function, progenitor cell, cell fate, generation, clonality

## Abstract

We consider the maintenance of ‘product’ cell populations from ‘progenitor’ cells via a sequence of one or more cell types, or compartments, where each cell’s fate is chosen stochastically. If there is only one compartment then large amplification, that is, a large ratio of product cells to progenitors comes with disadvantages. The product cell population is dominated by large families (cells descended from the same progenitor) and many generations separate, on average, product cells from progenitors. These disadvantages are avoided using suitably constructed sequences of compartments: the amplification factor of a sequence is the product of the amplification factors of each compartment, while the average number of generations is a sum over contributions from each compartment. Passing through multiple compartments is, in fact, an efficient way to maintain a product cell population from a small flux of progenitors, avoiding excessive clonality and minimizing the number of rounds of division *en route*. We use division, exit and death rates, estimated from measurements of single-positive thymocytes, to choose illustrative parameter values in the single-compartment case. We also consider a five-compartment model of thymocyte differentiation, from double-negative precursors to single-positive product cells.

## 1. Introduction

Cell populations in organs and tissues are continuously replenished. There are many biological systems in which a small flux of progenitor cells continuously replenishes large populations of ‘product’ cells via a structured developmental journey through a sequence of intermediate cell types [1–3]. Each cell type is referred to as a ‘compartment’, whether or not it corresponds to a physical location. In different contexts, product cells may be termed ‘mature’, ‘exhausted’, ‘fully differentiated’ or ‘effector’ cells [4–6]. We model such systems, assuming that cells in each compartment may die, divide or ‘transit’ to the next compartment, according to probabilistic rules. Only cells that reach the end of the sequence are called product cells. The set of product cells descended from a single progenitor is called a family. Theoretical and experimental arguments suggest that variability of family sizes is unavoidable if the fates of individual cells are subject to chance [7–10].

The dynamics of cellular developmental pathways is studied using recently developed heritable labels, where individual progenitor haematopoietic and immune cells are tagged and their progeny counted [9–12]. Different experimental definitions of what constitutes a compartment are adopted: most often, human or mouse cells are classified by the abundance of one or more types of molecules on their surface, measured using flow cytometry. For example, in a study of the specific CD8^+^ T-cell response to persistent *Toxoplasma gondii* infection, the surface markers CXCR3 and KLRG1 were used to identify an intermediate T-cell subset between memory and effector cells [13].

Maturation and selection of T cells in the thymus takes place via a sequence of cellular phenotypes, from bone-marrow progenitors to single-positive (SP4 or SP8) thymocytes [14–17], leading, in the case of an adult mouse, to about one million T cells per day exiting the thymus [18,19]. In an adaptive immune response, naive antigen-specific T-cell populations expand dramatically. The numbers and phenotypes of descendants of individual naive T cells are highly variable, but the magnitude of the total response is reproducible when the output of many families is combined [9,10,20]. Variability of family sizes is confirmed by direct time-lapse observations *in vitro* [8].

Hundreds of billions of blood cells are replaced every day in a typical adult, all descended from small numbers of haematopoietic stem cells (HSCs) [21–23]. HSCs produce multi-potent progenitor cells (MPPs) [2,10,24] through a hierarchy of cellular states [25]: more primitive HSC1s and more mature HSC2s, followed by MPP1, MPP2 and MPP3 cells. Low rates of division of cells in early compartments of a lineage are conjectured to reduce the risk that potentially cancerous mutations accumulate [26–28]. Increased risk of T-cell acute lymphoblastic leukaemia [29,30] is indeed found if the early compartments of the usual thymic sequence are absent [31,32].

Here, we examine the amplification of a small flux of progenitor cells to continuously replenish a product cell population from a theoretical perspective, based on stochastic rules governing the fates of individual cells. We calculate the probability distributions of the number of product cells per progenitor cell, and of the number of rounds of division that separates them. Our particular focus is on how these distributions depend on the number of compartments. Every cell in each compartment undergoes one of three fates: the cell may divide, die or make a transition to the next compartment [33–36]. The ‘transition’ event, corresponding to cell differentiation in many biological contexts, is called ‘exit’ for short. The balance of probabilities between fates depends on the compartment but each cell in a compartment chooses its fate independently. In this sense, our scheme is simpler than models that include interaction and competition between cells [37,38]. A consequence of our assumption of independence is that a cell’s division probability must be less than one half (otherwise the mean number of cells that descend from it would be infinite).

**Figure 1.**
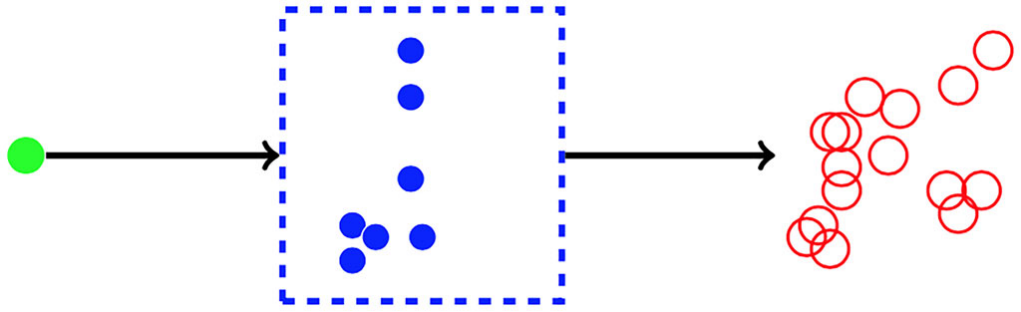
The one-compartment system. A single progenitor cell (shown on the left, green) is the founder of the population. In the compartment (represented by the dashed box), each cell (shown as a blue filled circle), independently, may die, divide or ‘exit’. An exit event is the differentiation of a cell to product cell type (shown as a red empty circle). The random variable **R** is the number of product cells when no cells remain in the compartment. We count the product cells as a cumulative total and do not consider any death or division events of product cells. The quantity *N* = IE(**R**) is the ‘amplification factor’: the mean number of product cells per progenitor.

**Figure 2.**
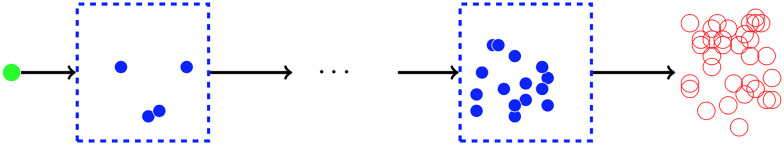
The multiple-compartment system. A single progenitor cell (shown on the left, green) is the founder of the population. Each cell in compartment *c*, independently, may die, divide or transit from compartment *c* to compartment *c* + 1, where *c* = 1, …, *C* − 1. Cells that exit compartment *C* are product cells (shown in red). The overall amplification factor *N* is the mean number of product cells per progenitor, which is the product of the amplification factors in each compartment.

We analyse the possible descendants of one progenitor cell, families of cells that journey through the sequence of compartments. The number of cells from one family that become product cells is the random variable **R**. To model the case where a small input flux of progenitors replenishes a larger product population, the mean of **R** will be large. In §2, we find the probability distribution of **R** as the ultimate state of a multi-type branching process [39]. The mean number of product cells per progenitor, IE(**R**), is denoted *N*. If there is a constant mean influx, *ϕ*, of progenitor cells, then there is a constant mean outflux, *Nϕ*, of product cells. The single-compartment case is illustrated in figure 1. It may be termed ‘direct differentiation’ because only one such event is needed to convert a progenitor cell to a product cell. We note that the product cell population (red circles) consists of cells that become product cells at different times. Similarly, the solid blue circles in figure 1 represent cells that are born, and may die, at different times. In this single-compartment scheme, large values of *N* are always associated with a high degree of clonality. Excessive ‘clonality’, where the variation in family size, from one progenitor to another, causes the population of product cells to be dominated by a few large families, may increase the risk of cancerous mutations becoming established in the population [40,41]. For example, the mean of **R** is equal to 10 if 10 per cent of progenitors yield 100 product cells, and the remainder yield none. One of our main results is that large values of *N* are possible without excessive clonality when the number of compartments, *C*, is greater than one, as illustrated in figure 2.

**Figure 3.**
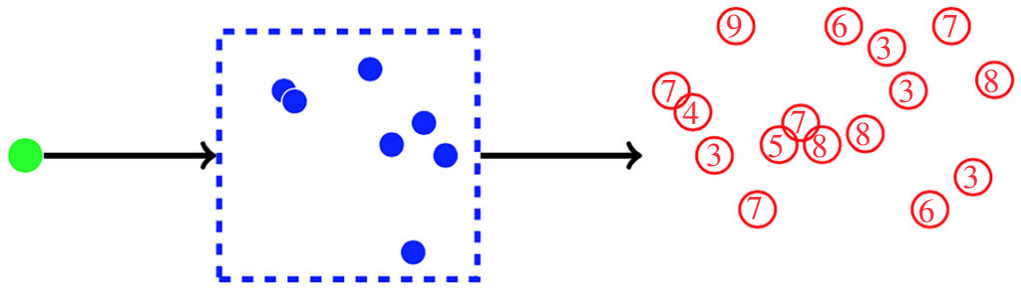
We classify the set of product (red) cells according to generation (number of divisions from the progenitor cell). The progenitor cell is said to be in generation 0. Whenever a cell in generation *n* divides, the result is two daughter cells in generation *n* + 1. The final state of the process is a population of red cells, each having made the transition at a different time and each with its own generation number. The case *C* = 1 is illustrated here. If *C* > 1 then the mean number of divisions in the product population is the sum of the mean numbers of divisions in each compartment.

The ability of product cells to perform their function may be negatively affected by the number of rounds of cell division that separates them from their progenitor, because every round of division brings with it a risk of mutation [42,43]. For this reason, as well as identifying an individual cell by the compartment it belongs to, *c* = 1, …, *C*, we label it by generation, *n* = 0, 1, …. The progenitor cell is said to be in generation 0. Whenever a cell in generation *n* divides, the result is two cells in generation *n* + 1 [44,45]. From this point of view, the population of product cells is heterogeneous because it is made up of cells of different generations (figure 3), cells with different ‘replicative histories’ [23] or ‘replicative ages’ [46]. Our analysis centres on the random variable **G**, defined to be the generation number of a randomly selected product cell.

The paper is organized as follows. Sections 2–4 consist of the main theoretical results and a set of remarks. In §2, we analyse the case *C* = 1. Explicit expressions for the distribution of family sizes are obtained via the probability generating function. In §3, we consider sequences of compartments: cells may make a transition from compartment *c* to compartment *c* + 1, for *c* = 1, …, *C* − 1. We treat the structured journey of development from a single progenitor cell to a population of product cells as a realization of a multi-type branching process [47,48]. By contrast, we note that discretized age-structured models [49] are different from sequences of compartments because birth events produce new individuals in the first compartment only. Since we are interested in the ultimate fate of the system, we proceed as in the theory of discrete-time branching processes, by defining relationships between random variables using probability generating functions. For instance, the probability generating function of the number of cells that exit the final compartment, descended from one progenitor cell, is given as a composition of probability generating functions. Note that, while mean quantities can also be obtained by solving linear systems of ordinary differential equations [50–54], the full distribution of **R** is encoded in its probability generating function. The product cell population, classified into generations, is examined in §4. In particular, we consider the random variable **G**: its mean value, *D*, and its distribution (as encoded in its probability generating function). In §5, we generalize our considerations to include a fourth type of event: asymmetric division; that is, a division event that leaves one daughter cell in the same compartment in which the mother cell divided and the other daughter cell exits the compartment. The appendices provide additional details, not included in the main body of the manuscript. In particular, the recursion relations that we use to generate the probability that *k* cells exit from one or two compartments are given in appendix A; the variance of the random variable **R**, which is proportional to *N*^2+1/*C*^ when *N* is large, is calculated in appendix B; and the generalization of our methods to include asymmetric division is presented in appendix C.

## 2. How many cells exit a compartment?

The case of one compartment is illustrated in figure 1. Three types of single-cell events contribute to the creation of a family of product cells from a single progenitor: individual cells may divide, die or transit (or differentiate) to a different cell type, or compartment. Our assumption is that every cell in a given compartment follows the same rules, independently, which is a fundamental assumption in branching processes [55,56]. Here, we restrict ourselves to counting cells, ignoring both inter-event times and the total time taken for progeny to disappear from all intermediate compartments and exit from the last one.

Analyses based on ordinary differential equations can calculate mean quantities, such as the mean number of product cells per progenitor. We, instead, calculate full distributions using first-step arguments and the probability generating function. The full distribution is of particular relevance in experiments where only a finite number of families can be tracked. When the rules at the level of a single cell are stochastic, some progenitors do not yield any product cells, while some found large families.

In this section, we analyse the case of one compartment, *C* = 1. Each cell in the compartment, independently, may die, divide or exit the compartment, with respective probabilities *p*_*d*_, *p*_*b*_ and *p*_*e*_, where *p*_*d*_ + *p*_*b*_ + *p*_*e*_ = 1. We assume that

2.1 $$p_{d}+p_{e}>p_{b},$$

so that extinction is the ultimate fate of the population of (blue) cells in the compartment. Exit has the same effect as death on the population in the compartment because exited cells play no further part in the dynamics of that compartment. Although the ultimate fate of the system is not affected by the inter-event time distributions, it is useful to keep in mind some examples that satisfy the assumptions that every cell independently, dies, divides or exits with probabilities *p*_*d*_, *p*_*b*_ and *p*_*e*_, respectively.

— A continuous-time birth–death-migration Markov process with exponential waiting times, where the probabilities *p*_*b*_, *p*_*d*_ and *p*_*e*_ are related to the rates of death, division and exit (i.e. migration), *μ*, *λ* and *ν*, respectively, by
  2.2 $$\begin{array}{c} p_{d}=\frac{\mu }{\mu +\nu +\lambda },\;p_{b}=\frac{\lambda }{\mu +\nu +\lambda }\;\mathrm{and}\\ \;p_{e}=\frac{\nu }{\mu +\nu +\lambda }. \end{array}$$
  Sawicka *et al.* [14] estimated *μ*, *λ* and *ν* for SP4 and SP8 thymocytes based on experimental data [57]. The estimated division rates were *λ*_4_ = 0.181 day^−1^ and *λ*_8_ = 0.085 day^−1^; death rates *μ*_4_ = 0.040 day^−1^ and *μ*_8_ = 0.110 day^−1^; and exit rates *ν*_4_ = 0.231 day^−1^ and *ν*_8_ = 0.152 day^−1^, respectively for SP4 and SP8 (see §3.3, table 2 of [14]).
— A population in which each cell is assigned three independent random variables: a death time *τ*_*d*_, a division time *τ*_*b*_ and a differentiation time *τ*_*e*_. The fate of the cell is whichever is the minimum of the three times [8,58]. Then, probabilities can be defined as follows:
  $$\begin{array}{rl} & p_{d}=P(\tau_{d}<\tau_{b}\text{ and }\tau_{d}<\tau_{e}),\\ \; & p_{b}=P(\tau_{b}<\tau_{d}\text{ and }\tau_{b}<\tau_{e}),\\ \text{and}\;\; & p_{e}=P(\tau_{e}<\tau_{b}\text{ and }\tau_{e}<\tau_{d}). \end{array}$$
  We note that (2.2) holds in the case where the probability densities of *τ*_*d*_, *τ*_*b*_ and *τ*_*e*_ are exponential.

The random variable **R** is the total number of product cells, starting from a single progenitor cell. Let us define *q*_*k*_ as follows:

2.3 $$q_{k}=P(R=k),\;k=0,1,2,\ldots .$$

We make use of the following argument based on the first event that occurs in the compartment. If the first event is cell division, then the two daughter cells, independently, follow the same rules as their mother. Therefore, *q*_0_ satisfies the quadratic equation

2.4 $$q_{0}=p_{d}+p_{b}q_{0}^{2}.$$

We can read (2.4) as a sum over the three possible first events, making use of the law of total probability

$$\sum_{s\in \{d,e,b\}}p_{s}\;P(R=0|\text{ first event is }s)=p_{d}1+p_{e}0+p_{b}q_{0}^{2}.$$

Because *q*_0_ is a probability, we take the solution of (2.4) in the interval [0, 1], given by

2.5 $$q_{0}=\frac{1-\Delta }{2p_{b}}=\frac{2p_{d}}{1+\Delta },\;\text{where}\;\Delta^{2}=1-4p_{d}p_{b}.$$

Similarly, the mean of **R** can be written as

2.6 $$N=IE(R)=\sum_{s\in \{d,e,b\}}p_{s}\;IE(R|\text{ first event is }s)=p_{d}0+p_{e}1+p_{b}2N,$$

so

2.7 $$N=\frac{\;p_{e}}{1-2p_{b}}.$$

The condition (2.1), which is equivalent to 2*p*_*b*_ < 1, assures that *N* is finite. We also observe that *p*_*b*_ must be close to $\frac{1}{2}$ for *N* to be large.

The probability *q*_1_ satisfies an equation similar to (2.4)

2.8 $$q_{1}=p_{e}+p_{b}2q_{0}q_{1}.$$

Thus, we have *q*_1_ = *p*_*e*_/Δ. We may find further *q*_*k*_ (for *k* ≥ 2) making use of the relationship

2.9 $$\begin{array}{c} q_{k}=p_{b}(q_{k}q_{0}+q_{k-1}q_{1}+\cdots +q_{1}q_{k-1}+q_{0}q_{k}),\;\text{so}\\ \;q_{k}=\frac{\;p_{b}}{\Delta }\sum_{\;j=1}^{k-1}q_{j}q_{k-j},\;k\geq 2. \end{array}$$

However, it is more convenient to consider the probability generating function of the random variable **R**, defined as

2.10 $$\phi (z)=IE(z^{R})=q_{0}+q_{1}z+q_{2}z^{2}+\cdots .$$

The probability generating function, like *q*_0_, satisfies a quadratic equation [59,60]

$$\phi (z)=\sum_{s\in \{d,e,b\}}p_{s}\;IE(z^{R}|\text{ first event is }s)=p_{d}z^{0}+p_{e}z^{1}+p_{b}\phi^{2}(z).$$

Thus, taking the sign of the square root that yields *ϕ*(1) = 1, we obtain

2.11 $$\phi (z)=\frac{1-{\left(1-4p_{b}p_{d}-4p_{b}p_{e}z\right)}^{1/2}}{2p_{b}}.$$

Using either (2.11) or (2.9), we find

2.12 $$q_{k}={\left(\frac{\;p_{b}}{\Delta }\right)}^{k-1}{\left(\frac{\;p_{e}}{\Delta }\right)}^{k}c_{k-1},\;k\geq 1,$$

where *c*_0_ = 1 and for *k* ≥ 1, we have

$$c_{k}=\frac{(2k)!}{k!(k+1)!}.$$

**Figure 4.**
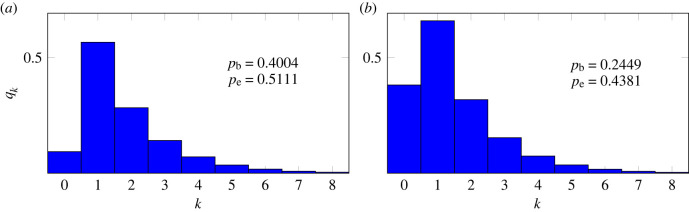
The quantity *q*_*k*_ is the probability that *k* cells exit a compartment, descended from one progenitor cell. Results, using (2.12), are shown for two different choices of *p*_*b*_ and *p*_*e*_. (*a*) We use the estimates of Sawicka *et al.* [14]: *p*_*b*_ = 0.4004 and *p*_*d*_ = 0.0885 for SP4 thymocytes. (*b*) Their estimates for SP8 thymocytes: *p*_*b*_ = 0.2449 and *p*_*d*_ = 0.3170.

The *c*_*k*_ are known as the Catalan numbers [61]. Examples of *q*_*k*_ are shown in figure 4 for two different choices of *p*_*b*_ and *p*_*e*_. With the estimates of Sawicka *et al.* [14], *N* ≃ 2.57 (for SP4 thymocytes) and *N* ≃ 0.86 (for SP8 thymocytes).

The distribution (2.12) of the random variable **R** is not one of the well-known distributions, such as Poisson or geometric. We therefore provide some remarks on its properties.

**Remark 2.1.**

Given any two of *p*_*d*_, *p*_*b*_ and *p*_*e*_, we can recover the third using *p*_*d*_ + *p*_*b*_ + *p*_*e*_ = 1. In fact, we may parametrize the compartment in terms of any two, linearly independent, combinations of *p*_*d*_, *p*_*b*_ and *p*_*e*_. We will, on occasions, use *N* itself along with *p*_*d*_. That is, using *N* = *p*_*e*_/(1 − 2*p*_*b*_), we can write

2.13 $$p_{b}=\frac{N-1+p_{d}}{2N-1}\;\text{and}\;p_{e}=\frac{N(1-2p_{d})}{2N-1}.$$

**Remark 2.2.**

The variance, *V*, of **R** is given by

2.14 $$V=\phi^{\prime \prime }(1)+N-N^{2}=\frac{2p_{b}}{p_{e}}N^{3}+N-N^{2},$$

which can be rewritten as

2.15 $$V=\frac{2}{1-2p_{d}}(N-1+p_{d})N^{2}+N-N^{2}.$$

Thus, the standard deviation of **R** is proportional to *N*^3/2^ as *N* → +∞.

**Remark 2.3.**

It is convenient to generate values of *q*_*k*_, (*k* ≥ 1), via the recursion relation

2.16 $$q_{k+1}=\frac{2k-1}{k+1}\frac{2p_{b}p_{e}}{1-4p_{b}p_{d}}q_{k}.$$

**Remark 2.4.**

We note that [62]

2.17 $$q_{k}<\frac{\;p_{e}}{\sqrt{\pi }\Delta }\gamma_{1}^{k-1}k^{-3/2},\;k\geq 1,$$

where we have introduced

2.18 $$\gamma_{1}=\frac{4p_{b}p_{e}}{1-4p_{b}p_{d}}.$$

If *N*≫1, then we have *γ*_1_ ≃ 1 − ((1 − 2*p*_*d*_)/4*N*^2^).

**Remark 2.5.**

The factor *k*^−3/2^ in (2.17) can be understood [63–65] as resulting from the square-root singularity in the probability generating function (2.11) rearranged as follows:

2.19 $$2p_{b}\phi (z)=1-\Delta {\left(1-\gamma_{1}z\right)}^{1/2}.$$

**Remark 2.6.**

The right-hand side of (2.17) is the asymptotic form of *q*_*k*_ as *k* → +∞ [62]. That is, we have

$$\log\left(\frac{q_{k+1}}{q_{k}}\right)≃\log\gamma_{1}-\frac{3}{2}\log\left(1+\frac{1}{k}\right),$$

when *k*≫ 1. If, in addition, *N*≫1 then we can write

2.20 $$\log\left(\frac{q_{k+1}}{q_{k}}\right)≃-\frac{1-2p_{d}}{4N^{2}}-\frac{3}{2}\frac{1}{k}.$$

**Figure 5.**
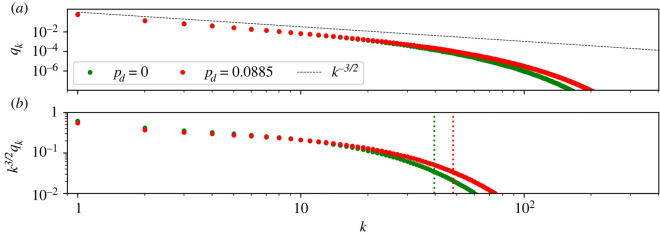
(*a*) The probability, *q*_*k*_ (using (2.12) and (2.16)), that the number of product cells is *k*, logarithmic scales, with and without death. The dashed line is the power law *q*_*k*_ = *k*^−3/2^. (*b*) *k*^3/2^*q*_*k*_ in the same two cases. The vertical dotted lines, at *k* = 6*N*^2^/(1 − 2*p*_*d*_), indicate where the power law ceases to be an accurate approximation. The parameter values, calculated using (2.13) so that *N* = 2.57 in both cases, are *p*_*d*_ = 0, *p*_*b*_ = 0.455, *p*_*e*_ = 0.545, and *p*_*d*_ = 0.0885, *p*_*b*_ = 0.4004, *p*_*e*_ = 0.5111. The latter set of values corresponds to those of SP4 thymocytes, as discussed above.

The decrease in *q*_*k*_ as a function of *k* is primarily due to the factor *k*^−3/2^, when (1 − 2*p*_*d*_)*k* < 6*N*^2^; thereafter, it is due to the factor $\gamma_{1}^{k}$ (figure 5). We may summarize the behaviour of *q*_*k*_ as having two regimes: it is first governed by the power law when *k* is small enough that $\gamma_{1}^{k}≃1$, then by the geometric term at values of *k* greater than 6*N*^2^/(1 − 2*p*_*d*_).

**Remark 2.7.**

In a population of cells made up of multiple realizations of **R**, we can also understand the dominance of large families of cells by evaluating *k*_50_, the lowest value of *k* such that half of the cells are part of a family of fewer than *k* cells. That is,

$$\frac{N}{2}<\sum_{k=1}^{k_{50}}kq_{k}.$$

Using (2.17), $kq_{k}<(\;p_{e}/\sqrt{\pi }\Delta )(1/\sqrt{k})$, so we can write

2.21 $$\begin{array}{cc} \; & \frac{\sqrt{\pi }\Delta }{2p_{e}}N<\sum_{k=1}^{k_{50}}\frac{1}{\sqrt{k}},\;\\ \; & \frac{\sqrt{\pi }\Delta }{2p_{e}}N<2\sqrt{k_{50}}\;\\ \mathrm{and}\; & k_{50}>\frac{\pi \Delta^{2}}{16p_{e}^{2}}N^{2}.\; \end{array}$$

Assuming *N* > 1 and using (2.13), we conclude

2.22 $$k_{50}>\frac{\pi }{16}\frac{\Delta^{2}}{{\left(1-2p_{d}\right)}^{2}}{\left(2N-1\right)}^{2}.$$

The factor Δ^2^/(1 − 2*p*_*d*_)^2^ is an increasing function of *p*_*d*_. In summary, for a given value of *N*, *k*_50_ is minimized by setting *p*_*d*_ = 0. An analytical bound on this minimum is *k*_50_ > (*π*/16)(2*N* − 1)^2^. Some numerical examples are: when *N* = 10 and *p*_*d*_ = 0, *k*_50_ = 83 and the analytical bound (2.22) is *k*_50_ > 71; when *N* = 10^2^ and *p*_*d*_ = 0, *k*_50_ = 9009 and the bound is *k*_50_ > 7775.

## 3. How many cells exit a sequence of compartments?

We now consider the case where there are *C* compartments before the final population of product cells. The random variable **R** is the number of product cells, descended from one cell in the first compartment. That is, there are *C* ‘transition’ or ‘differentiation’ events between the progenitor and the product phenotype. The case *C* = 1 was analysed in §2. The case *C* ≥ 2 is illustrated in figure 2.

Each cell, independently, may die, divide or make a transition from its current compartment to the next, with probabilities

$$p_{d}(c),\;p_{b}(c)\;\text{and}\;p_{e}(c),$$

where *p*_*d*_(*c*) + *p*_*b*_(*c*) + *p*_*e*_(*c*) = 1 for each *c*, with *c* = 1, …, *C*. The condition (2.1), that guarantees a finite number of product cells, is imposed in each compartment

$$p_{d}(c)+p_{e}(c)>p_{b}(c),\;\text{ for each }c,\;\text{with}\;c=1,\ldots ,C.$$

The quantity *N*_*c*_ = *p*_*e*_(*c*)/(1 − 2*p*_*b*_(*c*)) is the mean number of cells exiting compartment *c* for each cell that makes a transition to that compartment (from compartment *c* − 1). If **R**_*c*_ is the number of cells exiting compartment *c*, descended from one cell in compartment *c*, then the probability generating function of **R**_*c*_ is

3.1 $$\phi_{c}(z)=\frac{1-{\left[\Delta_{c}^{2}-4p_{b}(c)p_{e}(c)z\right]}^{1/2}}{2p_{b}(c)},\;c=1,\ldots ,C,$$

with $\Delta_{c}^{2}=1-4p_{d}(c)p_{b}(c)$. We can write *N*_*c*_ = IE(**R**_*c*_) = *ϕ*′_*c*_(1).

We seek *Q*_*k*_(*C*), the probability that the number of product cells, descended from a single progenitor via *C* intermediate compartments, is equal to *k*. We can write

3.2 $$Q_{k}(C)=P(R=k),\;k=0,1,2,\ldots .$$

The probability generating function of **R** is given by

3.3 $$\Phi_{C}(z)=IE(z^{R})=Q_{0}(C)+zQ_{1}(C)+z^{2}Q_{2}(C)+\cdots .$$

If *C* = 1 (there is only one compartment) then we recover the results of §1. That is, *Q*_*k*_(1) = *q*_*k*_ and $\Phi_{1}(z)=\phi_{1}(z)$. If *C* = 2, we may write

3.4 $$R=\sum_{i=1}^{R_{1}}R_{2,i},$$

where the **R**_2,*i*_ are identical and independent random variables with the same distribution as **R**_2_. Using (3.4), we find [55,56,60]

3.5 $$\Phi_{2}(z)=\phi_{1}(\phi_{2}(z)).$$

In general, we have

3.6 $$\Phi_{C}(z)=\phi_{1}(\phi_{2}(\cdots \phi_{C}(z))).$$

We maintain the notation that **R** is the number of product cells, *N* the mean and *V* the variance of **R**. The overall amplification factor is then given by

3.7 $$N=\prod_{c=1}^{C}N_{c}.$$

**Remark 3.1.**

The definition (3.3) relates the probability generating function to a set of probabilities. Different algorithms exist for extracting numerical values of the probabilities in situations where the probability generating function is known [66]. Because we have found it convenient to generate values of *Q*_*k*_(*C*) using a recursion relation similar to (2.16), we show how to obtain such relations in appendix A.

**Remark 3.2.**

An interesting feature of the distribution of **R** is the universality of its large-*k* behaviour

3.8 $$Q_{k}(C)\propto \gamma_{C}^{k}k^{-3/2},\;\text{as}\;k\to +\infty .$$

We may determine *γ*_*C*_ by locating the square-root singularity of $\Phi_{C}(z)$ [63–65]. We find that $\gamma_{1}=4p_{b}(1)p_{e}(1)/\Delta_{1}^{2}$ and *γ*_2_ satisfies $4p_{b}(1)p_{e}(1)\phi_{2}(\gamma_{2}^{-1})=\Delta_{1}^{2}$.

We define

3.9 $$\chi_{C}(z)=\phi_{2}(\phi_{3}(\cdots \phi_{C}(z))),$$

so that (2.19) is generalized to

3.10 $${\left[1-2p_{b}(1)\Phi_{C}(z)\right]}^{2}=\Delta_{1}^{2}[1-\gamma_{1}\chi_{C}(z)].$$

We expand around *z* = 1, making use of the fact that *χ*(1) = 1 and *χ*′(1) = *N*/*N*_1_, to obtain

$$\begin{array}{rl} & 1-\gamma_{1}\chi_{C}(z)≃1-\gamma_{1}\left[1-(1-z)\frac{N}{N_{1}}\right]\\ & \;=\left[\gamma_{1}\left(\frac{N}{N_{1}-1}\right)+1\right]\left(1-\frac{\gamma_{1}N/N_{1}}{\gamma_{1}(N/N_{1}-1)+1}z\right). \end{array}$$

We are then able to identify

3.11 $$\gamma_{C}={\left(1+\frac{1-\gamma_{1}}{\gamma_{1}N/N_{1}}\right)}^{-1}.$$

If *N*_1_, *N* ≫ 1 then $1-\gamma_{1}≃1/4N_{1}^{2}$ and we can approximate *γ*_*C*_ by the following expression:

3.12 $$\gamma_{C}≃1-\frac{1-2p_{d}(1)}{4N_{1}N}.$$

**Remark 3.3.**

If *C* > 2, we may make further progress with some assumptions to reduce the number of parameters. For example, consider the case where *N*_*c*_ is independent of *c* and *p*_*d*_(*c*) = 0 in each compartment. Then

— the variance of **R** is proportional to *N*^2+(1/*C*)^ as *N* → +∞ (for details, see appendix B), and
— the constant *γ*_*C*_ can be written as follows:
  3.13 $$\gamma_{C}=1-\frac{1}{4}\frac{1}{N^{1+1/C}}+\frac{1}{16}\frac{1}{N^{2(1+1/C)}}+\cdots .$$

**Figure 6.**
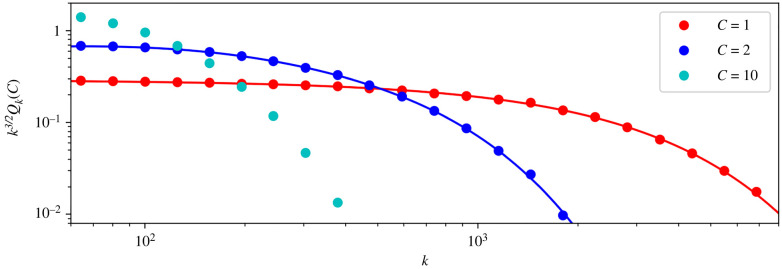
Plot of *k*^3/2^*Q*_*k*_(*C*) as a function of *k*, with logarithmic scales, for *C* = 1, *C* = 2 and *C* = 10. The distribution of **R** narrows as the number of compartments increases. The solid lines are the exact results, computed using (2.16) and (A 10). The dots are averages obtained from Gillespie realizations. Parameter values, chosen using (2.13) with *N* = 25, are *C* = 1: *p*_*d*_ = 0, *p*_*b*_ = 0.4898; *C* = 2: *p*_*d*_(1) = *p*_*d*_(2) = 0, *p*_*b*_(1) = *p*_*b*_(2) = 0.4444 and *N*_1_ = *N*_2_ = 5; *C* = 10: *p*_*d*_(*c*) = 0, *p*_*b*_(*c*) = 0.2158 and *N*_*c*_ = 1.38 for each *c* = 1, …, 10.

Figure 6 shows *k*^3/2^*Q*_*k*_(*C*) as a function of *k*, with parameters chosen as just described above. In all three cases shown, the mean number of product cells, *N*, is equal to 25 and *N*_*c*_ is independent of *c*. We shall see, below, that this choice of parameters is optimal from the perspective of minimizing the mean number of divisions per cell. The fact that the most efficient arrangement of compartments is found when each has the same amplification factor does not rule out different dynamics in different compartments. Indeed, a common scenario in cell biology is each compartment has faster rates than its predecessor [67–69].

**Remark 3.4.**

One effect of the presence of multiple compartments can be understood by comparison with the *k*_50_ values in remark 2.7 (for a single compartment). If *C* = 2, *N* = 10 and *p*_*d*_ = 0, then the *k*_50_ value is 33; if *C* = 2, *N* = 100 and *p*_*d*_ = 0, it is 1010. The corresponding *k*_50_ values when *C* = 3 are 25 and 528, for *N* = 10 and *N* = 100, respectively.

## 4. The population of exiting cells: how many divisions?

The progenitor cell is in generation 0. Daughter cells of the progenitor cell are said to be in generation 1. Daughter cells of a cell in generation *n* are in generation *n* + 1. In this way, the product cell population is classified by generation number, which is the number of divisions that separates a cell from the progenitor, or the depth of the cell in the tree that begins with the progenitor [70]. In §§2 and 3, we calculated the distribution of **R**, the number of product cells per progenitor, its mean and variance. In this section, we derive the probability generating function of the random variable **G**, the generation number of a randomly selected product cell.

### 4.1. Classifying cells by generation: a single compartment

To define the random variable **G**, we begin with two simple random variables, **U** and **V**, with state space {0, 2} and {0, 1}, respectively, and such that

$$\begin{array}{c} P(U=0)=1-p_{b},\;P(U=2)=p_{b}\;\text{and}\\ \;P(V=0)=1-p_{e},\;P(V=1)=p_{e}. \end{array}$$

We recall the random variables of a discrete-time branching process [55,56,71]. Let us introduce **Z**_0_ = 1 and

4.1 $$Z_{n+1}=\sum_{i=1}^{Z_{n}}U_{i},\;n=0,1,2,\ldots ,$$

where, for each *i*, **U**_*i*_ is an independent copy of **U**. **Z**_*n*_ is the number of cells in generation *n*, whatever their fate, and each **U**_*i*_ is the number of daughter cells from one cell. Here, we also need to define

4.2 $$Y_{n}=\sum_{i=1}^{Z_{n}}V_{i},\;n=0,1,2,\ldots ,$$

where each **V**_*i*_ is an independent copy of **V**. **Y**_*n*_ is the number of product cells in generation *n*. The random variables **R** and **G** are defined via

4.3 $$R=\sum_{n=0}^{+\infty }Y_{n}\;\text{and}\;P(G=n)=\frac{1}{N}IE(Y_{n}).$$

**Figure 7.**
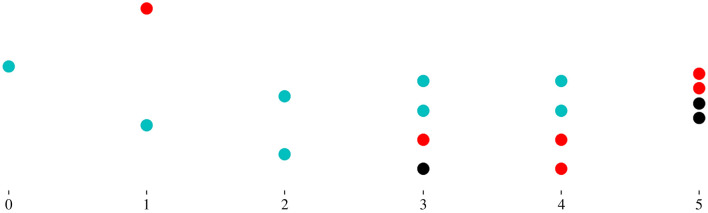
One realization with *C* = 1, showing generation numbers from left to right, with **Z**_0_ = 1. Cyan cells divide, red cells exit and black cells die. In this realization **Y**_0_ = 0, **Y**_1_ = 1, **Y**_2_ = 0, **Y**_3_ = 1, **Y**_4_ = 2 and **Y**_5_ = 2. Thus, we have **R** = 6. The parameter values are *p*_*b*_ = 0.45 and *p*_*d*_ = 0.15.

One realization of the process is shown in figure 7.

The mean values of **Y**_*n*_ are given by

4.4 $$IE(Y_{n})=p_{e}IE(Z_{n})=p_{e}{\left(2p_{b}\right)}^{n}.$$

The condition (2.1) is equivalent to 2*p*_*b*_ < 1. Hence, as *n* → +∞, $E(Z_{n})\to 0$ and $E(Y_{n})\to 0$.

Recall that the average number of product cells is *N* = *p*_*e*_/(1 − 2*p*_*b*_). The average generation number in the product cell population is given by

4.5 $$D=IE(G)=\frac{\;p_{e}}{N}\sum_{n=1}^{+\infty }n{\left(2p_{b}\right)}^{n}=\frac{2p_{b}}{1-2p_{b}}.$$

Using (4.3), we find that the variance of **G** is given by var(**G**) = *D*(*D* + 1).

**Figure 8.**
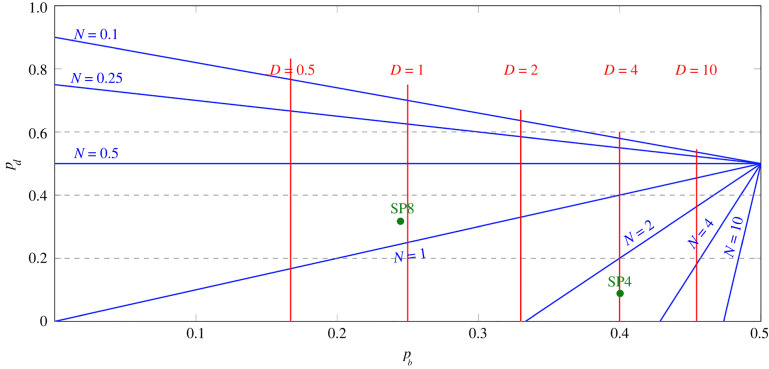
Lines of constant *D* (red) and lines of constant *N* (blue) in the part of the plane representing possible parameter values. The two quantities characterizing the population of cells exiting a compartment, as functions of *p*_*b*_ and *p*_*d*_, (2.7) and (4.5). Each blue line is the set of pairs (*p*_*b*_, *p*_*d*_) corresponding to the indicated value of *N*. Each red line is the set of pairs (*p*_*b*_, *p*_*d*_) corresponding to the indicated value of *D*. The triangular part of parameter space corresponding to *N* > 1 is at bottom right. The green dots are the estimates of Sawicka *et al.* [14], for SP4 and SP8 thymocytes.

In figure 8, *N* and *D* are displayed as functions of *p*_*b*_ and *p*_*d*_: lines of constant *N* are blue and lines of constant *D* are red. Also shown (in green) are the estimates of Sawicka *et al.* [14]: *p*_*b*_ = 0.4004 and *p*_*d*_ = 0.0885 (SP4 thymocytes) and *p*_*b*_ = 0.2449 and *p*_*d*_ = 0.3170 (SP8 thymocytes). We note the following limits: (i) as $p_{b}\to \frac{1}{2}$ with *p*_*d*_ fixed, *D*/*N* → 2/(1 − 2*p*_*d*_); (ii) as *p*_*b*_ → 0 with *p*_*d*_ fixed, *N* → 1 − *p*_*d*_ and *D* → 0.

**Remark 4.1.**

As in §2, we make use of the freedom to express all single-compartment quantities in terms of *N* and *p*_*d*_. Combining (2.6) and (4.5) gives the following linear relationship between *D* and *N*:

4.6 $$D=\frac{2N-1}{1-2p_{d}}-1.$$

Given *N* > 1, the minimum possible value of *D* is found when *p*_*d*_ = 0:

4.7 $$D_{\min}=2(N-1).$$

**Remark 4.2.**

We may express all single-compartment quantities in terms of variables which can be experimentally measured, such as number of product cells and generations, *N* and *D*. In particular, we have

$$p_{b}=\frac{1}{2}\frac{D}{D+1}\;\text{and}\;p_{e}=\frac{N}{D+1}.$$

These relationships could enable *p*_*b*_, *p*_*d*_ and *p*_*e*_ to be determined from experimentally measurable quantities, *N* = IE(**R**) and *D* = IE(**G**) [9,10,20]. The corresponding variances have simple expressions: *V* = var(**R**) = *N*^2^(*D* − 1) + *N* and var(**G**) = *D*(*D* + 1), respectively.

### 4.2. Classifying cells by generation: a sequence of *C* compartments

Cells that transit from compartment *c* to compartment *c* + 1, with *c* = 1, …*C* − 1, retain their generation number. Cells that exit compartment *C* are product cells. To analyse the multi-compartment system, we define the following sets of random variables, **Z**_*n*_(*c*) and **Y**_*n*_(*c*), as follows:

— For *n* ≥ 0 and 1 ≤ *c* ≤ *C*, **Z**_*n*_(*c*) is the number of generation *n* cells in compartment *c*, whatever their fate. We assume that **Z**_0_(1) = 1.
— For *n* ≥ 0 and 1 ≤ *c* ≤ *C*, **Y**_*n*_(*c*) is the number of generation *n* cells that exit compartment *c*. That is, **Y**_*n*_(*c*) ≤ **Z**_*n*_(*c*).

Then

$$Z_{0}(c)=Y_{0}(c-1),\;c=2,\ldots ,C.$$

To express the relationships between the random variables **Z**_*n*_(*c*) and **Y**_*n*_(*c*), we introduce for 1 ≤ *c* ≤ *C*, the random variables **U**(*c*) and **V**(*c*), with state space {0, 2} and {0, 1}, respectively, such that

$$\begin{array}{rl} & P(U(c)=0)=1-p_{b}(c),\;P(U(c)=2)=p_{b}(c)\\ \text{and}\; & P(V(c)=0)=1-p_{e}(c),\;P(V(c)=1)=p_{e}(c). \end{array}$$

The relation (4.1), standard in branching processes, is generalized to one that may appear in a branching process with immigration. For *c* ≥ 2, we have $Z_{n+1}(1)=\sum_{i=1}^{Z_{n}(1)}U_{i}(1)$ and

4.8 $$Z_{n+1}(c)=Y_{n+1}(c-1)+\sum_{i=1}^{Z_{n}(c)}U_{i}(c),\;c=2,\ldots ,C,\;n=0,1,\ldots$$

and

4.9 $$Y_{n}(c)=\sum_{i=1}^{Z_{n}(c)}V_{i}(c),\;c=1,\ldots ,C,\;n=0,1,\ldots$$

The number of product cells is the number of cells exiting the final compartment

4.10 $$R=\sum_{n=0}^{+\infty }Y_{n}(C).$$

**Figure 9.**
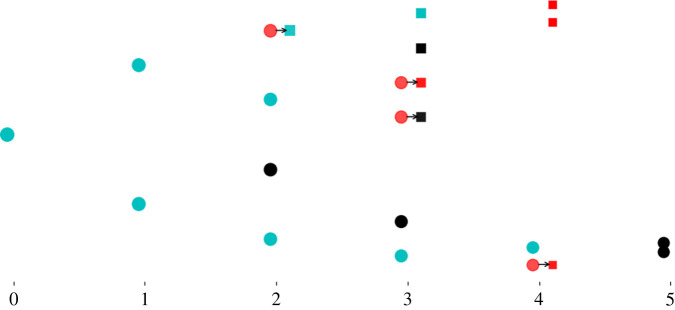
One realization with *C* = 2, showing generation numbers from left to right. Cells in the first compartment are shown as circles, and cells in the second compartment as squares. Cyan cells divide, red cells exit and black cells die. Arrows indicate a transition from the first to the second compartment. In this realization **Y**_0_(1) = 0, **Y**_1_(1) = 0, **Y**_2_(1) = 1, **Y**_3_(1) = 2, **Y**_4_(1) = 1 and **Y**_5_(1) = 0; **Y**_0_(2) = 0, **Y**_1_(2) = 0, **Y**_2_(2) = 0, **Y**_3_(2) = 1, **Y**_4_(2) = 3 and **Y**_5_(2) = 0. Thus, we have **R** = 4. The parameter values are *C* = 2, *p*_*b*_(1) = *p*_*b*_(2) = 0.45 and *p*_*d*_(1) = *p*_*d*_(2) = 0.15.

A realization of the multi-compartment process is illustrated in figure 9. The random variable **G** is the generation number of a randomly selected product cell

4.11 $$P(G=n)=\frac{1}{N}IE(Y_{n}(C)).$$

We consider the two mean quantities that characterize each compartment

4.12 $$\begin{array}{r} N_{c}=\frac{\;p_{e}(c)}{1-2p_{b}(c)}\;\text{and}\;D_{c}=\frac{2p_{b}(c)}{1-2p_{b}(c)},\;c=1,\ldots ,C. \end{array}$$

Thus, *N*_*c*_ is the mean number of cells exiting compartment *c*, descended from a single cell in compartment *c*, while *D*_*c*_ is the average increase in the generation number in the compartment (the average number of divisions undergone). We now introduce the following probability generating functions (for details, see appendix C.2), to keep track of the increase in generation number in compartment *c*, for *c* = 0, 1, …, *C*

4.13 $$\xi_{c}(z)=\frac{\;p_{e}(c)}{N_{c}}\sum_{n=1}^{+\infty }{\left(2zp_{b}(c)\right)}^{n}=\frac{1-2p_{b}(c)}{1-2p_{b}(c)z}.$$

For the whole sequence of compartments, let *N* be the mean number of product cells for every progenitor cell, and *D* be the average generation number of a product cell. Then

4.14  N=IE(R)=N1N2⋯NCand D=IE(G)=D1+D2+⋯+DC.

**Figure 10.**
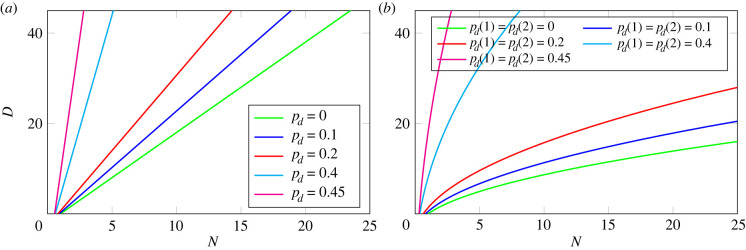
Average generation number of product cells, as a function of the mean number of exiting cells. (*a*) Plot for the case *C* = 1. (*b*) Plot for the case *C* = 2, with parameters chosen so that *N*_1_ = *N*_2_. Given a value of *N*, *D* is lower when *C* = 2 (proportional to $\sqrt{N}$ as *N* → +∞) than when *C* = 1 (proportional to *N* as *N* → +∞).

**Figure 11.**
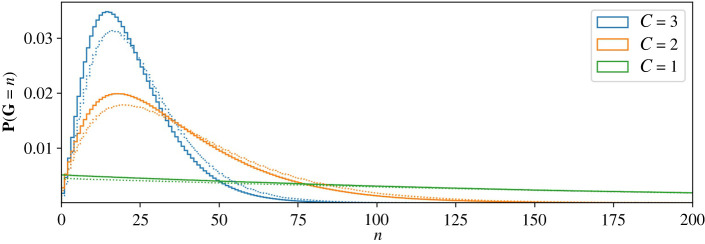
The probability distribution of the random variable **G**, the generation number in the product cell population. One, two and three compartments have been shown. In all cases, *N* = 100, and all compartments are identical. Solid lines correspond to *p*_*d*_ = 0 and dotted lines to *p*_*d*_ = 0.05.

The difference between a single compartment and a sequence of multiple compartments is already apparent if we compare *C* = 1 with *C* = 2, given the same value of *N*. In figure 10, we plot the average generation number, *D*, as a function of the mean number of exiting cells, *N*. In the examples with *C* = 2, shown on figure 10*b*, *N*_1_ = *N*_2_. The green lines show cases where there is no cell death. Given a value of *N*, *D* is lower when *C* = 2 (proportional to $\sqrt{N}$ as *N* → +∞) than when *C* = 1 (proportional to *N* as *N* → +∞). Figure 11 illustrates the probability distribution of **G** for different values of *C* with *N* fixed. The distribution narrows as the number of intermediate compartments increases.

Finally, the probability generating function of **G**, defined as $\Xi (z)=\sum_{n=0}^{+\infty }P(G=n)z^{n}$, is given by the product

4.15 $$\Xi (z)=\xi_{1}(z)\xi_{2}(z)\cdots \xi_{C}(z),$$

where, for each *c* = 1, …, *C*, *ξ*_*c*_(*z*) has been defined in (4.13).

### 4.3. Minimizing the average generation number

Since excessive ‘clonality’ may increase the risk of cancerous mutations becoming established [40,41], and because every round of division brings with it a risk of mutation, senescence or exhaustion [72–75], we now ask ourselves, how should a sequence of *C* compartments be constructed in order to yield a given amplification of progenitor to product cells, while minimizing the average number of divisions? Thus, given *N*, we seek to minimize *D*, given by (4.14). We write (4.12) as follows:

$$D_{c}=\alpha_{c}N_{c}-\beta_{c},\;\text{where}\;\alpha_{c}=\frac{2}{1-2p_{d}(c)}\;\text{and}\;\beta_{c}=\frac{2-2p_{d}(c)}{1-2p_{d}(c)}.$$

Let us imagine that the probabilities *p*_*d*_(*c*) are fixed, but the probabilities *p*_*b*_(*c*) are variable. Using the Lagrange multiplier method, we impose the constraint *N* = N* by defining

4.16 $$\begin{array}{rl} L(p_{b}(1),\ldots ,p_{b}(C),\Lambda ) & =D-\Lambda (N-N^{*})\\ & =\sum_{c=1}^{C}\frac{2p_{b}(c)}{1-2p_{b}(c)}\\ & \;-\Lambda \left(\prod_{c=1}^{C}\frac{1-p_{b}(c)-p_{d}(c)}{1-2p_{b}(c)}-N^{*}\right). \end{array}$$

We make use of the partial derivatives

$$\frac{\partial L}{\partial p_{b}(c)}=\frac{2}{{\left(1-p_{b}(c)\right)}^{2}}\left(1-\Lambda \frac{N^{*}}{\alpha_{c}N_{c}}\right),\;c=1,\ldots ,C,$$

to find the following conditions:

4.17 $$\alpha_{1}N_{1}=\alpha_{2}N_{2}=\cdots =\alpha_{C}N_{C}.$$

We continue the analysis by defining the arithmetic and geometric means of the *α*_*c*_

4.18 $$\bar{\alpha }=\frac{1}{C}\sum_{c=1}^{C}\alpha_{c}\;\text{ and }\;\tilde{\alpha }={\left(\prod_{c=1}^{C}\alpha_{c}\right)}^{1/C}.$$

Then, the optimal values of *N*_*c*_ have the property that

4.19 $$\alpha_{c}N_{c}=N^{1/C}\tilde{\alpha },\;\text{ for each}\;1\leq c\leq C.$$

The corresponding minimum value of *D* is then given by

4.20 $$D_{\min}=\sum_{c=1}^{C}(\alpha_{c}N_{c}-\beta_{c})=C\left(\tilde{\alpha }N^{1/C}-\frac{1}{2}\bar{\alpha }-1\right),$$

which is an increasing function of each of the *p*_*d*_(*c*) for 1 ≤ *c* ≤ *C*.

An interesting observation that can be made from the conditions (4.17) is that, if *p*_*d*_(*c*) does not depend on *c*, then *N*_*c*_ is also independent of *c*. That is, if the death probability does not vary from compartment to compartment, then the optimal arrangement of division rates is such that each compartment has the same amplification factor, *N*_*c*_ = *N*^1/*C*^. Then, we have

4.21 $$D_{\min}=\frac{2C}{1-2p_{d}}(N^{1/C}-1+p_{d}).$$

Given *N* and *C*, *D*_min_ is an increasing function of *p*_*d*_. We observe that *D*_min_ is a decreasing function of *C*. As *C* → +∞, *D*_min_ → 2log *N*, recovering the logarithmic behaviour characteristic of binary trees [42,76].

## 5. Asymmetric division

A subject of recent research is the possibility of asymmetric cell division, where one daughter cell remains in the mother’s compartment while the other transitions to the next compartment [9,38,46,77–83]. From the point of view of Markov processes, an asymmetric division event is unusual, in that division and change of cell type are supposed to be simultaneous. From a biological point of view, on the other hand, defining such an event may be natural: the mother’s intra-cellular and cell-surface proteins will not be exactly evenly partitioned between the two daughters, who may experience different conditions during the process of cell division [84,85]. From a modelling perspective, one could imagine the constant flux of progenitor cells in our scheme as being produced by a constant pool of stem cells undergoing asymmetric division.

The mathematics of asymmetric division is accommodated, as detailed in appendix C, by introducing a fourth type of event, asymmetric division, and its corresponding probability, *p*_*a*_. It is also possible to consider a fifth, where both daughter cells exit their mother’s compartment at birth [76], and to incorporate ‘de-differentiation’: cells moving backward in the hierarchy [86]. Böttcher *et al.* [46] developed a mathematical model with three types of event that all involve division: both daughter cells may remain in a compartment, both may transition, or one may remain and one transition. In this section, we explore and apply our methods to a biological system in which asymmetric cell division may play a role: T-cell development [81].

The development of thymocytes involves waves of proliferation, intertwined with differentiation, apoptosis and self-renewal to produce mature T cells, each with a unique T-cell receptor (TCR). T cell development takes place in the thymus and starts with lymphoid precursor cells, lacking expression of CD4 and CD8 co-receptors, known as double-negative (DN) thymocytes. The structured journey of development of these precursor cells involves the following stages, each of them defined by the cell-surface expression of developmentally regulated markers: DN1, DN2, DN3a, DN3b, DN4 and double-positive (DP) thymocytes [87,88]. Transition from the DN1 to DN2 stage marks the initiation of gene rearrangement at the TCR*β* gene locus [87]. The DN3 stage is characterized by the expression of the pre-T cell receptor (pre-TCR). It is at this stage that *β*-selection takes place; a checkpoint which defines the transition from the pre-selection DN3a to the post-selection DN3b stage. The DN3b population gives rise to the DN4 subset, which in turn undergoes proliferation and differentiation [88]. Further development involves the up-regulation of both CD4 and CD8 co-receptors to generate DP cells. Finally, DP cells go through gene rearrangement at the TCR*α* gene locus and the resulting *αβ* TCR heterodimer then undergoes major histocompatibility complex (MHC)-mediated selection to yield SP4 or SP8 cells.

**Figure 12.**
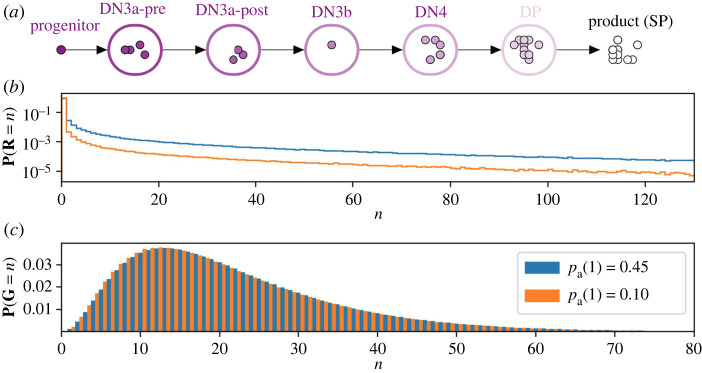
(*a*) Mathematical model of T cell development from the DN3a to the SP stage [81]. (*b*,*c*) Numerical results for two cases of the five-compartment thymus model. The histograms show the distributions of family sizes and of cell generation number in the population of product cells. The difference between the two cases is the first compartment, where only death and asymmetric division have non-zero probabilities. Table 1 gives the probabilities for all five compartments, and quantities derived from them.

Mammalian T-cell development suggests a possible role for asymmetric cell division [81] during the *β*-selection stage; subsequent divisions are predominantly symmetric. Pham *et al.* experimentally studied the DN3a to SP transition and defined a deterministic mathematical model of the process [81] (figure 12). Cells of the first compartment, DN3a-pre, can only die or undergo asymmetric cell division [81]. Thus, cells have already divided at least once when they arrive in the second compartment, as experimentally observed. The finding of Pham *et al.* that the death rate was larger than the rate of asymmetric division at the DN3a-pre stage implies, in the context of our model, that the probability of asymmetric cell division in the first compartment, *p*_*a*_(1), is smaller than $\frac{1}{2}$, with *p*_*a*_(1) + *p*_*d*_(1) = 1. Cells in compartments two (DN3a-post), three (DN3b), four (DN4) and five (DP) can die, divide (symmetrically) or differentiate (transition to the next compartment). We then write *p*_*b*_(*c*) + *p*_*d*_(*c*) + *p*_*e*_(*c*) = 1 for *c* = 2, 3, 4, 5. DN3 thymocytes undergo *β*-selection, which raises their probability of death. Accordingly, we choose *p*_*b*_(*c*) < *p*_*d*_(*c*) for DN3a-post and DN3b. By contrast, DN4 and DP thymocytes are more likely to divide than to die [87,88] (table 1).

**Table 1.** Parameter values for the five-compartment thymocyte development model. For any 1 ≤ *c* ≤ 5, *p*_*b*_(*c*) is the probability that a cell in compartment *c* divides, *p*_*d*_(*c*) is the probability that a cell in compartment *c* dies, *p*_*e*_(*c*) is the probability that a cell in compartment *c* transitions to compartment *c* + 1 and *p*_*a*_(*c*) is the probability that a cell in compartment *c* undergoes an asymmetric division event, where one daughter remains in compartment *c* and one transits to compartment *c* + 1. The values of *N*_*c*_ and *D*_*c*_ are calculated using (2.7) and (4.5).

	DN3a-pre	DN3a-post	DN3b	DN4	DP
*p*_*b*_ (*c*)	0	0.25	0.25	0.45	0.45
*p*_*e*_ (*c*)	0	0.3	0.3	0.3	0.3
*p*_*d*_ (*c*)	0.55/0.9	0.45	0.45	0.25	0.25
*p*_*a*_ (*c*)	0.45/0.1	0	0	0	0
*N*_*c*_	(9/11)/(1/9)	0.6	0.6	3	3
*D*_*c*_	(20/11)/(10/9)	1	1	9	9

The analysis of Pham *et al.* was purely deterministic and therefore only considered mean numbers of cells in each compartment. In figure 12, we show the distributions of two biologically significant random variables in our stochastic model: the number of product cells in a family founded by one progenitor and the generation number of a cell in the product cell (here, SP) population. Two cases are shown, *p*_*a*_(1) = 0.1 and *p*_*a*_(1) = 0.45. In the first, 90% of DN3a-pre cells die, so the average family size in the product population, *N* = 0.36, is smaller, on average, than in the second case, when only 55% of DN3a-pre cells die and *N* = 2.651. (These values are the product of the *N*_*c*_ values in table 1.) Nevertheless, in both cases families of over 10^2^ cells are not uncommon. Single-positive thymocytes are released from the thymus to the periphery, where families of cells correspond to TCR clonotypes [18,19,89–91]. In a mouse, where division of naive T cells in the periphery is rare, the diversity of the T-cell repertoire (the number of different TCRs simultaneously present) and the distribution of family sizes are determined by the distribution of family sizes at the time of release from the thymus [90–94].

The distributions of generation number **G** are also shown in figure 12. They are relatively narrow: product cells with **G** > 100 are rare. The difference between the distributions with *p*_*a*_(1) = 0.1 and *p*_*a*_(1) = 0.45 is small because, in both cases, the majority of cells that make the transition DN3a-pre to DN3a-post do so in the first generation. The mean values, *D* = 21.1 and *D* = 21.9, respectively, may be obtained by summing the values of *D*_*c*_, *c* = 1, …, 5 given in table 1.

In the example we have analysed in this section, the intermediate compartments have a rationale related to TCR selection that is independent of family sizes and the distribution of generation numbers: we may conclude nature has made a virtue of the necessity of passing through multiple stages. However, intermediate compartments are also found in other cellular replenishment systems without an obvious independent reason.

## 6. Conclusion

Cells of the same phenotype are often thought of as belonging to a compartment, which may correspond to a spatial location, a biological function, or simply a set of cell-surface attributes which can be measured with flow cytometry. In many circumstances, a population of ‘product’ cells performing a specific role is maintained, via a sequence of compartments, from a much smaller progenitor population. Why are multiple such compartments so often observed rather than a simpler one-step differentiation from progenitor to product cell? Using theoretical arguments, we show why such schemes are advantageous. In our model, individual cells in a compartment may die or divide (in the compartment), or transition to the next compartment, meaning that they change phenotype or ‘differentiate’. Our mathematical approach is based on two fundamental biological (or empirical) observations: amplification (from progenitor cell to product cell populations) and stochasticity (of the fate of individual cells). Thus, we assume that each cell in a given compartment, independently, chooses one of the available fates according to a shared set of probabilities: *p*_*b*_, *p*_*e*_ and *p*_*d*_ are the probabilities of division, transition and death, respectively. When a cell divides, its daughter cells, independently, follow the same rules as their mother. Hence, all population properties are deduced from a complete understanding of the possible progeny of a single progenitor. Furthermore, the population of product cells is the sum of families, each founded by a single progenitor cell. We do not consider inter-event times. Rather, each realization is a sequence of events that ultimately results in extinction of the progeny in the pre-product compartment or compartments, with only product cells surviving. We construct sequences of *C* compartments, where cells may transit from compartment *c* to compartment *c* + 1, with *c* = 1, …, *C* − 1. Given an overall amplification factor, *N*, the dominance of large families of cells in the product cell population decreases as *C* increases. Using probability generating functions, we find *Q*_*k*_(*C*), the probability that the number of product cells, descended from a single progenitor via *C* intermediate compartments, is equal to *k*. When *k* is large, $Q_{k}(C)\propto \gamma_{C}^{k}k^{-3/2}$, with *γ*_*C*_ < 1.

Our model deals in probabilities, which we relate to two important quantities, *N* and *D*, that can be measured in some experiments. The first, *N*, is the average number of product cells descended from a single progenitor, which can be measured if the progenitor cell is given a heritable label. The second, *D*, is the mean generation number of the product cell population, which can be measured if progenitor cells are stained with a fluorescent dye that dilutes with division, such as cell trace CFSE or cell trace violet. A recently developed genetic tracing technique called *DivisionRecorder* makes it possible to measure the mean number of divisions of immune cell populations up to dozens of rounds of division [20]. The analysis presented in this manuscript shows that both *N* and *D* have long-tailed distributions when there are no intermediate compartments, and it allows us to quantify the reduction of clonality and long-term division history in product cell populations as the number of compartments is increased [95].

When there is only a single compartment (that is, when progenitor cells differentiate directly into product cells) the mean number of product cells per progenitor is related to an individual cell’s division and exit probabilities by *N* = *p*_*e*_/(1 − 2*p*_*b*_) and the mean generation number in the product cell population is given by *D* = 2*p*_*b*_/(1 − 2*p*_*b*_). Thus, large values of *N*, found when the value of *p*_*b*_ is less than but close to $\frac{1}{2}$, lead to large values of *D*. The presence of intermediate compartments is advantageous from this point of view: the mean generation number, *D*, decreases as *C* increases. Given *N*, the minimum value of *D*, found when *p*_*d*_ is zero, is given by *D*_min_ = 2*C*(*N*^1/*C*^ − 1). Whatever the value of *p*_*d*_, the most efficient arrangement of compartments is found when each has the same amplification factor.

Our theoretical analyses are found in §2 for a single compartment, §3 for a sequence of compartments and §4 for the number of divisions in the compartmental system. We find that a sequence of compartments achieves the amplification of progenitor to product cells required in tissue organization and homeostasis while avoiding excessive clonality and minimizing the average number of divisions. Section 5 applies our methods to the structured development journey of thymocytes, where we generalize our considerations to include asymmetric division; that is, a division event that leaves one daughter cell in the same compartment in which the mother cell divided and the other daughter cell exits the compartment. Additional details have been provided in the appendices: the recursion relations to obtain the probability that *k* cells exit from one or two compartments are given in appendix A; the variance of the random variable **R** is calculated in appendix B; and the generalization of our methods to include asymmetric division is presented in appendix C.

## Figures and Tables

**Figure 13.**
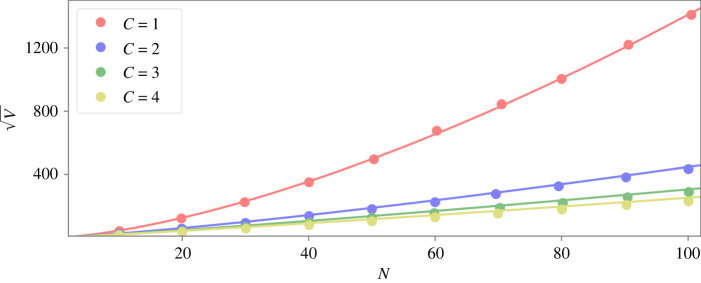
The standard deviation of **R** as a function of the mean of **R**, *N*, for different values of *C*. The lines use the formula (B 1), and each line corresponds to one value of *C*. The dots are obtained as averages over numerical realizations. Parameter values have been chosen so that *N*_*c*_ is independent of *c*, *p*_*d*_(*c*) = 0, and thus, *N*_*c*_ = *N*^1/*c*^, *p*_*e*_(*c*) = 1 − *p*_*b*_(*c*) and *p*_*b*_(*c*) = (*N*_*c*_ − 1)/(2*N*_*c*_ − 1), for all *c* = 1, …, *C*.

**Figure 14.**
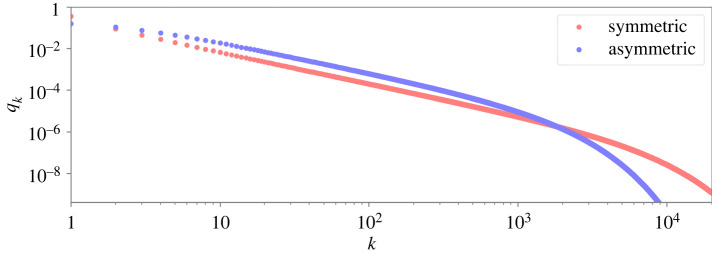
The distribution of **R**, with and without asymmetric division, when *C* = 1. In red, the symmetric case (2.12), *p*_*a*_ = 0, and in blue, the purely asymmetric case, *p*_*e*_ = 0, generated using (C 9). In both cases, we have chosen *N* = 25 and *p*_*d*_ = 0.25.

**Figure 15.**
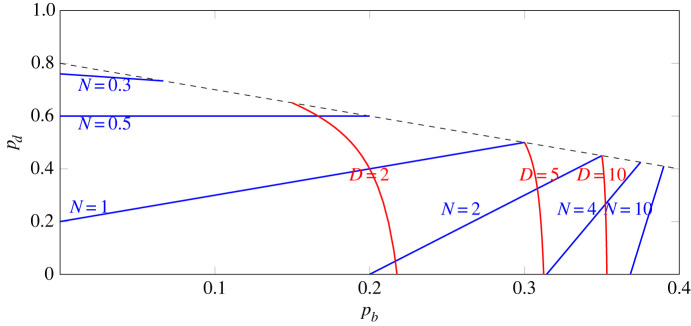
Lines of constant *N* (blue) and curves of constant *D* (red) in the part of the plane representing possible parameter values when *p*_*a*_ = 0.2. Each blue line is the set of pairs (*p*_*b*_, *p*_*d*_) corresponding to the indicated value of *N*. Each red curve is the set of pairs (*p*_*b*_, *p*_*d*_) corresponding to the indicated value of *D*.

## Data Availability

Python codes to perform Gillespie simulations to generate figure 6 (Qkhist.py), figure 11 (Gdist04.py) and figure 12 (RGdist06.py) are available at https://doi.org/10.5281/zenodo.7181108 [96].

## Appendix A. Recursion relation for the probabilities *Q*_*k*_(*C*)

In principle, the whole distribution of a random variable can be obtained once its probability generating function is known. In practice, an algorithm is required to compute the numerical values of the desired probabilities [66]. Here, we describe equations that we have used, relating the probability that the random variable **R** is equal to *k* to the probability that it is equal to *k* − 1, in the simplest case (*C* = 1), and to *k* − 1 and *k* − 2 in other cases (*C* = 2).

**A.1. Recursion relation: a single compartment**

In the case *C* = 1, we rewrite (2.11) as 2*p*_*b*_*ϕ*(*z*) = 1 − *w*(*z*), where *w*^2^(*z*) = 1 − 4*p*_*b*_*p*_*d*_ − 4*p*_*b*_*p*_*e*_*z*. We now compute the first derivative of *ϕ*(*z*). One can show that *w*(*z*)*ϕ*′(*z*) = *p*_*e*_ and that *ϕ*(*z*) satisfies the following differential equation:

A 1 $$w^{2}(z)\phi^{\prime }(z)+2p_{e}p_{b}\phi (z)-p_{e}=0.$$

Inserting $\phi (z)=\sum_{k=0}^{+\infty }q_{k}z^{k}$ in (A 1), and matching terms proportional to *z*^*k*^ yields the recursion relation (2.16).

**A.2. Recursion relation: two compartments**

We next consider the case *C* = 2. In what follows, we obtain a differential equation for $\Phi_{2}(z)$ of the form

A 2 $$T(z)\Phi_{2}^{\prime \prime }(z)+R(z)\Phi_{2}^{\prime }(z)+S(z)(1-2p_{b}(1)\Phi_{2}(z))=0,$$

with *T*(*z*), *R*(*z*) and *S*(*z*) polynomials in *z* (with real coefficients), and given by

$$T(z)=t_{0}+t_{1}z+t_{2}z^{2},\;R(z)=r_{0}+r_{1}z\;\text{and}\;S(z)=s_{0}.$$

Then, given that $\Phi_{2}(z)=\phi_{1}(\phi_{2}(z))=\sum_{k=0}^{+\infty }Q_{k}z^{k}$,

A 3 $$\begin{array}{rl} & t_{0}(k+2)(k+1)Q_{k+2}+[t_{1}k^{2}+(r_{0}+t_{1})k+r_{0}]Q_{k+1}\\ & \;+[t_{2}k^{2}+(r_{1}-t_{2})k+s_{0}]Q_{k}=0. \end{array}$$

Let us write $\Delta_{c}^{2}=1-4p_{d}(c)p_{b}(c)$ and $w_{c}^{2}(z)=\Delta_{c}^{2}-4p_{b}(c)$ $p_{e}(c)z$, for *c* = 1, 2. We have $2p_{b}(1)\Phi_{2}(z)=1-w_{1}(\phi_{2}(z))$ and

A 4 $$\Phi_{2}^{\prime }(z)=\frac{\;p_{e}(1)}{w_{1}}\phi_{2}^{\prime }(z)=\frac{\;p_{e}(1)p_{e}(2)}{w_{1}w_{2}},$$

where *w*_1_ is shorthand for *w*_1_(*ϕ*_2_(*z*)), and *w*_2_ is shorthand for *w*_2_(*z*). Now, we compute the second derivative of $\Phi_{2}(z)$

A 5 $$\Phi_{2}^{\prime \prime }(z)=\frac{2p_{e}(1)p_{e}^{2}(2)}{w_{1}^{3}w_{2}^{3}}[p_{b}(1)p_{e}(1)w_{2}+p_{b}(2)w_{1}^{2}].$$

Multiplying through by $w_{1}^{3}w_{2}^{3}$, we can write

A 6 $$\begin{array}{rl} & 2p_{e}(1)p_{e}^{2}(2)[p_{b}(2)w_{1}^{2}+p_{b}(1)p_{e}(1)w_{2}]T(z)\\ & \;+p_{e}(1)p_{e}(2)w_{2}^{2}w_{1}^{2}R(z)+w_{2}^{3}w_{1}^{4}S(z)=0. \end{array}$$

We make use of the fact that $1-2p_{b}(1)\Phi_{2}(z)=w_{1}$ and that $w_{1}^{2}=\Delta_{1}^{2}-\kappa +\kappa w_{2}$, where *κ* = 2*p*_*e*_(1)(*p*_*b*_(1)/*p*_*b*_(2)). Equating terms proportional to $w_{2}^{2}$, $w_{2}^{3}$, $w_{2}^{4}$ and $w_{2}^{5}$, we find

A 7 T(z)=T2w22+T4w24,R(z)=R0+R2w22and S(z)=s0=2pb(1)pe2(1)pe2(2),

where

$$\begin{array}{rl} & T_{2}=-{\left(\Delta_{1}^{2}-\kappa \right)}^{2},\;T_{4}=\kappa^{2},\;R_{0}=-2p_{b}(2)p_{e}(2)T_{2},\\ & R_{2}=-4p_{b}(2)p_{e}(2)T_{4}\;\text{and}\;s_{0}=2p_{b}(1)p_{e}^{2}(1)p_{e}^{2}(2). \end{array}$$

Making use of (A 7), we obtain

$$\begin{array}{rl} & t_{0}=\Delta_{2}^{2}T_{2}+\Delta_{2}^{4}T_{4},\;t_{1}=-4p_{b}(2)p_{e}(2)T_{2}-8p_{b}(2)p_{e}(2)\Delta_{2}^{2}T_{4},\\ & t_{2}=16p_{b}^{2}(2)p_{e}^{2}(2)T_{4},\;r_{0}=\frac{1}{2}t_{1}\;\text{and}\;r_{1}=t_{2}. \end{array}$$

The general two-compartment recursion relation (A 3) is thus given by

$$\begin{array}{rl} & [\kappa^{2}\Delta_{2}^{4}-{\left(\Delta_{1}^{2}-\kappa \right)}^{2}\Delta_{2}^{4}](k+1)(k+2)Q_{k+2}\\ & \;-p_{b}(2)p_{e}(2)[2\kappa^{2}\Delta_{2}^{2}-{\left(\Delta_{1}^{2}-\kappa \right)}^{2}](2k+1)(2k+2)Q_{k+1} \end{array}$$

A 8 $$\;+p_{b}^{2}(2)p_{e}^{2}(2)\kappa^{2}(16k^{2}-1)Q_{k}=0.$$

If *p*_*d*_(1) = *p*_*d*_(2) = 0, then Δ_1_ = Δ_2_ = 1 and (A 8) takes the simpler form

A 9 $$\begin{array}{rl} & \left(2\kappa -1)(k+1)(k+2)Q_{k+2}-p_{b}(2)p_{e}(2)(\kappa^{2}+2\kappa -1\right)\\ & \;(2k+1)(2k+2)Q_{k+1}+p_{b}^{2}(2)p_{e}^{2}(2)\kappa^{2}(16k^{2}-1)Q_{k}=0. \end{array}$$

## Appendix B. The variance of the distribution of family sizes

The distributions of family sizes that we have found have a pattern where the factor *k*^−3/2^ appears. One consequence of this behaviour is that the relationship between the mean and variance is different from that found in well-known distributions such as the Poisson distribution.

With *C* compartments, the probability generating function of **R** is given by (3.6), and the variance of **R** is given by

B 1 $$V=\Phi_{C}^{\prime \prime }(1)+N-N^{2}.$$

We make use of (3.9), to write $\Phi_{C}^{\prime }(z)=\phi_{1}^{\prime }(\chi_{C}(z))\chi_{C}^{\prime }(z)$ and $\Phi_{C}^{\prime \prime }(1)=\phi_{1}^{\prime \prime }(1){\left(\chi_{C}^{\prime }(1)\right)}^{2}+\phi_{1}^{\prime }(1)\chi_{C}^{\prime \prime }(1),$ where $\Phi_{C}^{\prime }(z)=$ $\left(d/\;dz)\Phi_{C}(z\right)$.

We next assume that *ϕ*_*c*_(*z*) = *ϕ*(*z*), *c* = 1, …, *C*. Then, one can show that

 ϕ′(1)=N1/C,ϕ″(1)=2 pbpeN3/Cand ΦC″(1)=2 pbpe[N3/CN2(1−1/C)]+N1/CχC″(1).

We find

 Φ1″(1)=2 pbpeN3,Φ2″(1)=2 pbpe(N5/2+N2)and Φ3″(1)=2 pbpe(N7/3+N2+N5/3),….

That is, we have

B 2 $$\Phi_{C}^{\prime \prime }(1)=2\frac{\;p_{b}}{p_{e}}N^{2+1/C}\sum_{c=0}^{C-1}N^{-c/C}.$$

The variance of **R** is proportional to *N*^2+(1/*C*)^ in the limit *N* → +∞ (figure 13).

## Appendix C. Compartment analysis in the case of asymmetric division

In an asymmetric division event, one daughter cell transits to the next compartment and the other remains in the compartment. Each cell, independently, may die, divide, undergo asymmetric division, or transit to the next compartment, with probabilities

$$p_{d},\;p_{b},\;p_{a}\;\text{and}\;p_{e},$$

where *p*_*d*_ + *p*_*b*_ + *p*_*e*_ + *p*_*a*_ = 1. The analogue of (2.1), guaranteeing extinction in the compartment, is

C 1 $$2p_{b}+p_{a}<1.$$

**C.1. Family sizes**

Proceeding to the calculation of the *q*_*k*_ as in §2, we find that (2.5) still holds, but (2.8) and (2.9) are replaced by Δ*q*_1_ = *p*_*e*_ + *p*_*a*_ *q*_0_ and

C 2 $$q_{k}=\frac{\;p_{b}}{\Delta }\sum_{\;j=1}^{k-1}q_{j}q_{k-j}+\frac{\;p_{a}}{\Delta }q_{k-1},\;k\geq 2.$$

The probability generating function of **R** when *C* = 1, denoted by *ψ*(*z*), satisfies

C 3 $$\psi (z)=p_{d}+p_{e}z+p_{a}z\psi (z)+p_{b}\psi^{2}(z).$$

The solution is given by

C 4 $$\psi (z)=\frac{1-p_{a}z-{\left[{\left(1-p_{a}z\right)}^{2}-4p_{b}p_{d}-4p_{b}p_{e}z\right]}^{1/2}}{2p_{b}}.$$

Figure 14 compares *q*_*k*_ in this case (asymmetric case) with that of symmetric division only (*p*_*a*_ = 0).

Thus, in the case of asymmetric division, and for *C* = 1, we have

C 5 $$N=\frac{\;p_{e}+p_{a}}{1-2p_{b}-p_{a}}.$$

Remark C.1. If $q_{k}=P(R=k)$ then, for *k* ≥ 2,

$$\begin{array}{rl} q_{k} & =\frac{\Delta }{p_{b}}{\left(\frac{2p_{b}q_{1}+p_{a}}{2\Delta }\right)}^{k}\sum_{\;j=0}^{\left\lfloor k/2\rfloor \right)}c_{k-j-1}\left(\begin{array}{c} k-j\\ j \end{array}\right){\left(\frac{-2p_{a}^{2}\Delta }{\left(2p_{a}+4p_{b}p_{e})(2p_{b}q_{1}+p_{a}\right)}\right)}^{j}\\ & =\frac{\Delta }{p_{b}}{\left(\frac{2p_{b}q_{1}+p_{a}}{2\Delta }\right)}^{k}\sum_{\;j=0}^{\left\lfloor k/2\right\rfloor }\frac{1}{k-j}\left(\begin{array}{c} 2k-2j-1\\ k-j \end{array}\right)\left(\begin{array}{c} k-j\\ j \end{array}\right)\\ & \;\times {\left(\frac{-2p_{a}^{2}\Delta }{\left(2p_{a}+4p_{b}p_{e})(2p_{b}q_{1}+p_{a}\right)}\right)}^{j}. \end{array}$$

Remark C.2. It is convenient to generate *q*_*k*_ via a recursion relation. Following the approach described in appendix A, we rewrite (C 5) as

C 6 $$\begin{array}{rl} & 2p_{b}\psi (z)=1-p_{a}z-w_{a}(z),\\ & \;\text{where}\;w_{a}^{2}(z)=\Delta -(2p_{a}+4p_{b}p_{e})z+p_{a}^{2}z^{2}. \end{array}$$

Thus, *ψ*(*z*) satisfies the following differential equation:

C 7 $$w_{a}^{2}(z)\psi^{\prime }(z)+w_{a}^{\prime }(z)w_{a}(z)\psi (z)+\zeta (z)=0,$$

with $\zeta (z)=(p_{a}^{2}-p_{a}+2p_{a}p_{b}p_{e}-4p_{b}p_{e})z+\Delta^{2}-p_{a}-2p_{b}p_{e}$. Matching terms proportional to *z*^*k*^ leads to the following recursion relation:

C 8 $$\Delta^{2}(k+2)q_{k+2}=(2k+1)(p_{a}+2p_{b}p_{e})q_{k+1}-(k-1)p_{a}^{2}q_{k}.$$

We note that in the asymmetric case, even for *C* = 1, the recursion relation is of second order. This is due to the fact that $w_{a}^{2}(z)$ is a polynomial of order two in *z*.

Remark C.3. As *k* → +∞, we obtain the following behaviour:

C 9 $$q_{k}\propto \gamma_{a}^{k}k^{-3/2},$$

where *γ*_*a*_ satisfies the equation

C 10 $$(1-4p_{b}p_{d})\gamma_{a}^{2}-(2p_{a}+4p_{b}p_{e})\gamma_{a}+p_{a}^{2}=0.$$

Remark C.4. We now consider the case *C* = 2, with two non-identical compartments, i.e. *ψ*_1_(*z*) ≠ *ψ*_2_(*z*). Let us introduce

C 11 $$\Psi_{2}(z)=\psi_{1}(\psi_{2}(z))$$

and

C 12 $$\begin{array}{rl} w_{a,c}^{2}(z) & =1-4p_{b}(c)p_{d}(c)-[2p_{a}(c)+4p_{b}(c)p_{e}(c)]z\\ & \;+p_{a}^{2}(c)z^{2},\;c=1,2. \end{array}$$

Then, one can show that

C 13 $$2p_{b}(1)\Psi_{2}(z)=H_{1}(z)-H_{2}(z),$$

where *H*_1_(*z*) = 1 − (*p*_*a*_(1)/2*p*_*b*_(2)) + (*p*_*a*_(1)*p*_*a*_(2)/2*p*_*b*_(2)) − (*p*_*a*_(1)/2*p*_*b*_(2))*w*_*a*2_(*z*), and

$$\begin{array}{rl} H_{2}{\left(z\right)}^{2} & =\frac{\;p_{a}^{2}(1)p_{a}^{2}(2)}{2p_{b}^{2}(2)}z^{2}+\left(\frac{\;p_{a}(2)}{p_{b}(2)}(p_{a}(1)+2p_{b}(1)p_{e}(1)-\frac{\;p_{a}^{2}(1)}{p_{b}^{2}(2)}(p_{a}(2)+p_{b}(2)p_{e}(2))\right)z\\ & \;+\left(\frac{\;p_{a}(1)+2p_{b}(1)p_{e}(1)}{p_{b}(2)}-\frac{\;p_{a}^{2}(1)(1-p_{a}(2)z)}{2p_{b}^{2}(2)}\right)w_{a,2}(z)\\ & \;+\Delta^{2}(1)+\frac{\;p_{a}^{2}(1)}{2p_{b}^{2}(2)}(1-2p_{d}(2)p_{b}(2))-\frac{\;p_{a}(1)+2p_{b}(1)p_{e}(1)}{p_{b}(2)}. \end{array}$$

In this instance, for the asymmetric case with *C* = 2, and to calculate the distribution of probabilities, *Q*_*k*_(2), we must compute two recursion relations: one for *H*_1_(*z*) and a second one for *H*_2_(*z*). This strategy leads to a three-term recursion relation for *H*_1_(*z*), and a six-term recursion relation for *H*_2_(*z*).

**C.2. Generation analysis**

To define the random variable **G**, we begin with three simple random variables **U**, **V** and **W**, with state spaces {0, 1, 2}, {0, 1} and {0, 1}, respectively, where

$$P(U=0)=1-p_{b}-p_{a},\;P(U=1)=p_{a},\;P(U=2)=p_{b}$$

and

$$P(V=0)=1-p_{e},\;P(V=1)=p_{e}\;\text{and}\;P(W=0)=1-p_{a},\;P(W=1)=p_{a}.$$

Let us introduce, as we did in the case of symmetric division, **Z**_0_ = 1 and

C 14 $$Z_{n+1}=\sum_{i=1}^{Z_{n}}U_{i},\;n=0,1,2,\ldots ,$$

where, for each *i*, **U**_*i*_ is an independent copy of **U** (as defined above). The definition (4.1) still holds, but (4.2) is replaced by

C 15 $$Y_{n}=\sum_{i=1}^{Z_{n}}V_{i}+\sum_{i=1}^{Z_{n-1}}W_{i},\;n=0,1,2,\ldots ,$$

where each **V**_*i*_, **W**_*i*_ are, respectively, an independent copy of **V** and **W**. The mean values of **Y**_*n*_ for *n* ≥ 0, which generalize (4.4), are given by IE(**Y**_0_) = *p*_*e*_ and

$$IE(Y_{n})=p_{e}E(Z_{n})+p_{a}E(Z_{n-1})=p_{e}{\left(2p_{b}+p_{a}\right)}^{n}+p_{a}{\left(2p_{b}+p_{a}\right)}^{n-1}.$$

Once again, and due to condition (C 2), in the limit *n* → +∞, IE(**Z**_*n*_) → 0 and IE(**Y**_*n*_) → 0. We are interested in obtaining the probability generating function of **G**. Making use of the definition of the random variables **R** and **G**, the probability generating function of **G** is given by

C 16 $$\xi (z)=\frac{1}{N}\sum_{n=0}^{+\infty }IE(Y_{n})z^{n}=\frac{1}{N}\left[\;p_{e}+\sum_{n=1}^{+\infty }IE(Y_{n})z^{n}\right]=\frac{\;p_{e}+p_{a}z}{N(1-(2p_{b}+p_{a})z)}.$$

This allows us to compute the expectation value of **G** in the asymmetric case

C 17 $$IE(G)=D=\frac{1}{p_{e}+p_{a}}\frac{\;p_{a}+p_{e}(2p_{b}+p_{a})}{1-2p_{b}-p_{a}}.$$

The variance of **G** is also computed from (C 17)

C 18 $$\mathrm{var}(G)=\frac{2p_{a}+p_{e}}{p_{e}}D(D+1).$$

Remark C.5. In the case of asymmetric division, we can choose *N*, *p*_*a*_ and *p*_*d*_ as the three independent parameters, so that (4.6) is given by

C 19 $$D=\frac{2N-1}{1+p_{a}-2p_{d}}\left(1+\frac{\;p_{a}}{N}\right)-1.$$

Figure 15, constructed using (C 18), summarizes the effect of asymmetric division (when compared with figure 8).

Remark C.6. We may express all single-compartment quantities in terms of *N*, *D* and *p*_*a*_, to obtain

 pb=N[D(1−pa)−pa]−pa2N(D+1),pe=N−paDD+1and pd=N[2+D(1+pa)−2N−pa]+pa2N(D+1).

Note that, if we set *p*_*a*_ = 0 then all quantities simplify to the values derived in §4.

Remark C.7. In the case of *C* > 1 compartments, and asymmetric division, we define for c=1,…,C the following random variables **U**(*c*), **V**(*c*) and **W**(*c*):

$$\begin{array}{rl} & P(U(c)=0)=1-p_{b}(c)-p_{a}(c),\;P(U(c)=1)=p_{a}(c),\;\\ & P(U(c)=2)=p_{b}(c),P(V(c)=0)=1-p_{e}(c),\;\\ & P(V(c)=1)=p_{e}(c)\;\text{and}\;P(W(c)=0)=1-p_{a}(c),\;\\ & P(W(c)=1)=p_{a}(c). \end{array}$$

We have in this case **Z**_0_(1) = 1, **Z**_0_(*c*) = **Y**_0_(*c* − 1) for *c* ≥ 2, and

C 20 $$\begin{array}{rl} & Z_{n+1}(c)=Y_{n+1}(c-1)+\sum_{i=1}^{Z_{n}(c)}U_{i}(c),\;c=2,\ldots ,C,\\ & \;n=0,1,2,\ldots \end{array}$$

and

C 21 $$\begin{array}{rl} & Y_{n}(c)=\sum_{i=1}^{Z_{n}(c)}V_{i}(c)+\sum_{i=1}^{Z_{n-1}(c)}W_{i}(c),\;c=2,\ldots ,C,\\ & \;n=1,2,\ldots . \end{array}$$

The probability generating function of **G** is given by a product of single-compartment generating functions making use of (4.15).

## References

[1] Busch K, Klapproth K, Barile M, Flossdorf M, Holland-Letz T, Schlenner SM, Reth M, Höfer T, Rodewald H-R. 2015 Fundamental properties of unperturbed haematopoiesis from stem cells in vivo. Nature **518**, 542-546. (10.1038/nature14242) 25686605

[2] Höfer T, Barile M, Flossdorf M. 2016 Stem-cell dynamics and lineage topology from *in vivo* fate mapping in the hematopoietic system. Curr. Opin Biotechnol. **39**, 150-156. (10.1016/j.copbio.2016.04.001) 27107166

[3] Sawai CM et al. 2016 Hematopoietic stem cells are the major source of multilineage hematopoiesis in adult animals. Immunity **45**, 597-609. (10.1016/j.immuni.2016.08.007) 27590115 PMC5054720

[4] Thomas-Vaslin HK, Altes HK, de Boer HK, Klatzmann D, 2008 Comprehensive assessment and mathematical modeling of T cell population dynamics and homeostasis. J. Immunol. **180**, 2240. (10.4049/jimmunol.180.4.2240) 18250431

[5] Johnston MD, Edwards CM, Bodmer WF, Maini PK, Chapman SJ. 2007 Mathematical modeling of cell population dynamics in the colonic crypt and in colorectal cancer. Proc. Natl Acad. Sci. USA **104**, 4008-4013. (10.1073/pnas.0611179104) 17360468 PMC1820699

[6] Robert PA, Kunze-Schumacher H, Greiff V, Krueger A. 2021 Modeling the dynamics of T-cell development in the thymus. Entropy **23**, 437. (10.3390/e23040437) 33918050 PMC8069328

[7] Till JE, McCulloch EA, Siminovitch L. 1964 A stochastic model of stem cell proliferation, based on the growth of spleen colony-forming cells. Proc. Natl Acad. Sci. USA **51**, 29. (10.1073/pnas.51.1.29) 14104600 PMC300599

[8] Duffy KR, Hodgkin PD. 2012 Intracellular competition for fates in the immune system. Trends Cell Biol. **22**, 457-464. (10.1016/j.tcb.2012.05.004) 22727035

[9] Gerlach C et al. 2013 Heterogeneous differentiation patterns of individual CD8+ T cells. Science **340**, 635-639. (10.1126/science.1235487) 23493421

[10] Perié L, Hodgkin PD, Naik SH, Schumacher TN, de Boer RJ, Duffy KR. 2014 Determining lineage pathways from cellular barcoding experiments. Cell Rep. **6**, 617-624. (10.1016/j.celrep.2014.01.016) 24508463

[11] Buchholz VR, Schumacher TNM, Busch DH. 2016 T cell fate at the single-cell level. Annu. Rev. Immunol. **34**, 65-92. (10.1146/annurev-immunol-032414-112014) 26666651

[12] Miles AS, Hodgkin PD, Duffy KR. 2021 Inferring differentiation order in adaptive immune responses from population-level data. In *Mathematical, computational and experimental T cell immunology*, pp. 133–149. Berlin, Germany: Springer.

[13] Chu HH, Chan S-W, Gosling JP, Blanchard N, Tsitsiklis A, Lythe G, Shastri N, Molina-París C, Robey EA. 2016 Continuous effector CD8^+^ T cell production in a controlled persistent infection is sustained by a proliferative intermediate population. Immunity **45**, 159-171. (10.1016/j.immuni.2016.06.013) 27421704 PMC4956557

[14] Sawicka M, Stritesky GL, Reynolds J, Abourashchi N, Lythe G, Molina-París C, Hogquist KA. 2014 From pre-DP, post-DP, SP4, and SP8 thymocyte cell counts to a dynamical model of cortical and medullary selection. Front. Immunol. **5**, 19. (10.3389/fimmu.2014.00019) 24592261 PMC3924582

[15] Krueger A, Zietara N, Łyszkiewicz M. 2017 T cell development by the numbers. Trends Immunol. **38**, 128-139. (10.1016/j.it.2016.10.007) 27842955

[16] Sinclair C, Bains I, Yates AJ, Seddon B. 2013 Asymmetric thymocyte death underlies the CD4:CD8 T-cell ratio in the adaptive immune system. Proc. Natl Acad. Sci. USA **110**, E2905-E2914. (10.1073/pnas.1304859110) 23858460 PMC3732981

[17] Yates A. 2014 Theories and quantification of thymic selection. Front. Immunol. **5**, 13. (10.3389/fimmu.2014.00013) 24550908 PMC3912788

[18] den Braber I et al. 2012 Maintenance of peripheral naive T cells is sustained by thymus output in mice but not humans. Immunity **36**, 288-297. 22365666 10.1016/j.immuni.2012.02.006

[19] Hogan T, Gossel G, Yates AJ, Seddon B. 2015 Temporal fate mapping reveals age-linked heterogeneity in naive T lymphocytes in mice. Proc. Natl Acad. Sci. USA **112**, E6917-E6926. (10.1073/pnas.1517246112) 26607449 PMC4687551

[20] Bresser K et al. 2022 Replicative history marks transcriptional and functional disparity in the CD8+ T cell memory pool. Nat. Immunol. **23**, 791-801. (10.1038/s41590-022-01171-9) 35393592 PMC7612726

[21] Abkowitz JL, Golinelli D, Harrison DE, Guttorp P. 2000 In vivo kinetics of murine hemopoietic stem cells. Blood **96**, 3399-3405. (10.1182/blood.V96.10.3399) 11071634

[22] Sender R, Milo R. 2021 The distribution of cellular turnover in the human body. Nat. Med. **27**, 45-48. (10.1038/s41591-020-01182-9) 33432173

[23] Cosgrove J, Hustin LSP, de Boer RJ, Perié L. 2021 Hematopoiesis in numbers. Trends Immunol. **42**, 1100-1112. (10.1016/j.it.2021.10.006) 34742656

[24] Becker NB, Günther M, Li C, Jolly A, Höfer T. 2019 Stem cell homeostasis by integral feedback through the niche. J. Theor. Biol. **481**, 100-109. (10.1016/j.jtbi.2018.12.029) 30579956

[25] Hofer T et al. 2020 Hematopoietic stem cells self-renew symmetrically or gradually proceed to differentiation. *bioRxiv*. (10.1101/2020.08.06.239186)

[26] Tomasetti C, Vogelstein B. 2015 Variation in cancer risk among tissues can be explained by the number of stem cell divisions. Science **347**, 78-81. (10.1126/science.1260825) 25554788 PMC4446723

[27] Tomasetti C, Li L, Vogelstein B. 2017 Stem cell divisions, somatic mutations, cancer etiology, and cancer prevention. Science **355**, 1330-1334. (10.1126/science.aaf9011) 28336671 PMC5852673

[28] Bowman RL, Busque L, Levine RL. 2018 Clonal hematopoiesis and evolution to hematopoietic malignancies. Cell Stem Cell **22**, 157-170. (10.1016/j.stem.2018.01.011) 29395053 PMC5804896

[29] Martins VC, Ruggiero E, Schlenner SM, Madan V, Schmidt M, Fink PJ, von Kalle C, Rodewald H-R. 2012 Thymus-autonomous T cell development in the absence of progenitor import. J. Exp. Med. **209**, 1409-1417. (10.1084/jem.20120846) 22778389 PMC3420332

[30] Ballesteros-Arias L, Silva JG, Paiva RA, Carbonetto B, Faísca P, Martins VC. 2019 T cell acute lymphoblastic leukemia as a consequence of thymus autonomy. J. Immunol. **202**, 1137-1144. (10.4049/jimmunol.1801373) 30651344

[31] Peaudecerf L, Lemos S, Galgano A, Krenn G, Vasseur F, Di Santo JP, Ezine S, Rocha B. 2012 Thymocytes may persist and differentiate without any input from bone marrow progenitors. J. Exp. Med. **209**, 1401-1408. (10.1084/jem.20120845) 22778388 PMC3420331

[32] Boehm T. 2012 Self-renewal of thymocytes in the absence of competitive precursor replenishment. J. Exp. Med. **209**, 1397-1400. (10.1084/jem.20121412) 22851642 PMC3420333

[33] Ogawa M. 1993 Differentiation and proliferation of hematopoietic stem cells. Blood **81**, 2844-2853. (10.1182/blood.V81.11.2844.2844) 8499622

[34] Abkowitz JL, Catlin SN, Guttorp P. 1996 Evidence that hematopoiesis may be a stochastic process in vivo. Nat. Med. **2**, 190-197. (10.1038/nm0296-190) 8574964

[35] Reya T, Morrison SJ, Clarke MF, Weissman IL. 2001 Stem cells, cancer, and cancer stem cells. Nature **414**, 105-111. (10.1038/35102167) 11689955

[36] Xu J, Wang Y, Guttorp P, Abkowitz JL. 2018 Visualizing hematopoiesis as a stochastic process. Blood Adv. **2**, 2637-2645. (10.1182/bloodadvances.2018023705) 30327372 PMC6199667

[37] Roeder I, Horn M, Glauche I, Hochhaus A, Mueller MC, Loeffler M. 2006 Dynamic modeling of imatinib-treated chronic myeloid leukemia: functional insights and clinical implications. Nat. Med. **12**, 1181-1184. (10.1038/nm1487) 17013383

[38] Grajzel D, Derényi I, Szöllősi GJ. 2020 A compartment size-dependent selective threshold limits mutation accumulation in hierarchical tissues. Proc. Natl Acad. Sci. USA **117**, 1606-1611. (10.1073/pnas.1913104117) 31907322 PMC6983402

[39] Tak T, Prevedello G, Simon G, Paillon N, Benlabiod C, Marty C, Plo I, Duffy KR, Perié L. 2021 HSPCs display within-family homogeneity in differentiation and proliferation despite population heterogeneity. Elife **10**, e60624. (10.7554/eLife.60624) 34002698 PMC8175087

[40] Wainscoat JS, Fey MF. 1990 Assessment of clonality in human tumors: a review. Cancer Res. **50**, 1355-1360. 1967978

[41] Lyne A-M, Laplane L, Perié L. 2021 To portray clonal evolution in blood cancer, count your stem cells. Blood **137**, 1862-1870. (10.1182/blood.2020008407) 33512426

[42] Kay HEM. 1965 How many cell-generations? Lancet **286**, 418-419. (10.1016/S0140-6736(65)90763-4) 14348601

[43] Werner B, Dingli D, Traulsen A. 2013 A deterministic model for the occurrence and dynamics of multiple mutations in hierarchically organized tissues. J. R. Soc. Interface **10**, 20130349. (10.1098/rsif.2013.0349) 23740488 PMC4043170

[44] Samuels ML. 1971 Distribution of the branching-process population among generations. J. Appl. Probab. **8**, 655-667. (10.2307/3212230)

[45] Meli G, Weber TS, Duffy KR. 2019 Sample path properties of the average generation of a Bellman–Harris process. J. Math. Biol. **79**, 673-704. (10.1007/s00285-019-01373-0) 31069504

[46] Böttcher MA, Dingli D, Werner B, Traulsen A. 2018 Replicative cellular age distributions in compartmentalized tissues. J. R. Soc. Interface **15**, 20180272. 30158183 10.1098/rsif.2018.0272 PMC6127166

[47] Antal T, Krapivsky PL. 2011 Exact solution of a two-type branching process: models of tumor progression. J. Stat. Mech: Theory Exp. **2011**, P08018. (10.1088/1742-5468/2011/08/P08018)

[48] Luria SE, Delbrück M. 1943 Mutations of bacteria from virus sensitivity to virus resistance. Genetics **28**, 491. (10.1093/genetics/28.6.491) 17247100 PMC1209226

[49] Caswell H. 2000 Matrix population models: construction, analysis, and interpretation, vol. 1, 2nd edn. Sunderland, MA: Sinauer Associates Inc.

[50] Zierler K. 1981 A critique of compartmental analysis. Annu. Rev. Biophys. Bioeng. **10**, 531-562. (10.1146/annurev.bb.10.060181.002531) 7259129

[51] Colijn C, Mackey MC. 2005 A mathematical model of hematopoiesis—I. Periodic chronic myelogenous leukemia. J. Theor. Biol. **237**, 117-132. (10.1016/j.jtbi.2005.03.033) 15975596

[52] Michor F, Hughes TP, Iwasa Y, Branford S, Shah NP, Sawyers CL, Nowak MA. 2005 Dynamics of chronic myeloid leukaemia. Nature **435**, 1267-1270. (10.1038/nature03669) 15988530

[53] Whichard ZL, Sarkar CA, Kimmel M, Corey SJ. 2010 Hematopoiesis and its disorders: a systems biology approach. Blood **115**, 2339-2347. (10.1182/blood-2009-08-215798) 20103779 PMC2845894

[54] Werner B, Dingli D, Lenaerts T, Pacheco JM, Traulsen A. 2011 Dynamics of mutant cells in hierarchical organized tissues. PLoS Comput. Biol. **7**, e1002290. (10.1371/journal.pcbi.1002290) 22144884 PMC3228763

[55] Harris TE. 1963 The theory of branching processes. Berlin, Germany: Springer.

[56] Kimmel M, Axelrod DE. 2002 Branching processes in biology. Berlin, Germany: Springer.

[57] Stritesky GL, Xing Y, Erickson JR, Kalekar LA, Wang X, Mueller DL, Jameson SC, Hogquist KA. 2013 Murine thymic selection quantified using a unique method to capture deleted T cells. Proc. Natl Acad. Sci. USA **110**, 4679-4684. (10.1073/pnas.1217532110) 23487759 PMC3606987

[58] Marchingo JM, Prevedello G, Kan A, Heinzel S, Hodgkin PD, Duffy KR. 2016 T-cell stimuli independently sum to regulate an inherited clonal division fate. Nat. Commun. **7**, 1-12. (10.1038/ncomms13540) PMC5121331 27869196

[59] Steel JM. 2001 Stochastic calculus and financial applications. Berlin, Germany: Springer.

[60] Wilf HS. 2005 Generatingfunctionology. New York, NY: CRC Press.

[61] Singmaster D. 1978 An elementary evaluation of the Catalan numbers. Am. Math. Mon. **85**, 366-368. (10.1080/00029890.1978.11994597)

[62] Dutton RD, Brigham RC. 1986 Computationally efficient bounds for the Catalan numbers. Eur. J. Comb. **7**, 211-213. (10.1016/S0195-6698(86)80024-5)

[63] Knuth DE, Wilf HS. 1989 A short proof of Darboux’s lemma. Appl. Math. Lett. **2**, III-IV. (10.1016/0893-9659(89)90007-4)

[64] Greene DH, Knuth DE. 1990 Mathematics for the analysis of algorithms. Boston, MA: Birkhauser.

[65] Flajolet P, Sedgewick R. 2009 Analytic combinatorics. Cambridge, UK: Cambridge University Press.

[66] Gleeson JP, Ward JA, O’Sullivan KP, Lee WT. 2014 Competition-induced criticality in a model of meme popularity. Phys. Rev. Lett. **112**, 048701. (10.1103/PhysRevLett.112.048701) 24580496

[67] Kaech SM, Wherry EJ, Ahmed R. 2002 Effector and memory T-cell differentiation: implications for vaccine development. Nat. Rev. Immunol. **2**, 251-262. (10.1038/nri778) 12001996

[68] Hasbold J, Corcoran LM, Tarlinton DM, Tangye SG, Hodgkin PD. 2004 Evidence from the generation of immunoglobulin G-secreting cells that stochastic mechanisms regulate lymphocyte differentiation. Nat. Immunol. **5**, 55-63. (10.1038/ni1016) 14647274

[69] Höfer T, Rodewald H-R. 2018 Differentiation-based model of hematopoietic stem cell functions and lineage pathways. Blood, J. Am. Soc. Hematol. **132**, 1106-1113. 10.1182/blood-2018-03-791517 PMC6307983 30042097

[70] Duffy KR, Meli G, Shneer S. 2019 The variance of the average depth of a pure birth process converges to 7. Stat. Probab. Lett. **150**, 88-93. (10.1016/j.spl.2019.02.015)

[71] Stirzaker D. 2005 Stochastic processes and models. Oxford, UK: Oxford University Press.

[72] Partridge L, Gems D. 2002 Mechanisms of aging: public or private? Nat. Rev. Genet. **3**, 165-175. (10.1038/nrg753) 11972154

[73] Mathon NF, Lloyd AC. 2001 Cell senescence and cancer. Nat. Rev. Cancer **1**, 203-213. (10.1038/35106045) 11902575

[74] Philip M, Schietinger A. 2021 CD8+ T cell differentiation and dysfunction in cancer. Nat. Rev. Immunol. **22**, 209-223. (10.1038/s41577-021-00574-3) 34253904 PMC9792152

[75] Akbar AN, Henson SM. 2011 Are senescence and exhaustion intertwined or unrelated processes that compromise immunity? Nat. Rev. Immunol. **11**, 289-295. (10.1038/nri2959) 21436838

[76] Derényi I, Szöllősi GJ. 2017 Hierarchical tissue organization as a general mechanism to limit the accumulation of somatic mutations. Nat. Commun. **8**, 1-8. 28230094 10.1038/ncomms14545 PMC5331224

[77] Werner B, Beier F, Hummel S, Balabanov S, Lassay L, Orlikowsky T, Dingli D, Brümmendorf TH, Traulsen A. 2015 Reconstructing the in vivo dynamics of hematopoietic stem cells from telomere length distributions. eLife **4**, e08687. (10.7554/eLife.08687) 26468615 PMC4744200

[78] Yang J, Plikus MV, Komarova NL. 2015 The role of symmetric stem cell divisions in tissue homeostasis. PLoS Comput. Biol. **11**, e1004629. (10.1371/journal.pcbi.1004629) 26700130 PMC4689538

[79] Stiehl T, Marciniak-Czochra A. 2017 Stem cell self-renewal in regeneration and cancer: insights from mathematical modeling. Curr. Opin. Syst. Biol. **5**, 112-120. (10.1016/j.coisb.2017.09.006)

[80] Shahriyari L, Komarova NL. 2013 Symmetric vs. asymmetric stem cell divisions: an adaptation against cancer? PLoS ONE **8**, e76195. (10.1371/journal.pone.0076195) 24204602 PMC3812169

[81] Pham K et al. 2015 Asymmetric cell division during T cell development controls downstream fate. J. Cell Biol. **210**, 933-950. (10.1083/jcb.201502053) 26370500 PMC4576854

[82] Barile M et al. 2020 Hematopoietic stem cells self-renew symmetrically or gradually proceed to differentiation. Available at *SSRN 3787896*. (10.2139/ssrn.3787896)

[83] Flossdorf M, Rössler J, Buchholz VR, Busch DH, Höfer T. 2015 CD8+ T cell diversification by asymmetric cell division. Nat. Immunol. **16**, 891-893. (10.1038/ni.3235) 26287584

[84] Chang JT et al. 2007 Asymmetric T lymphocyte division in the initiation of adaptive immune responses. Science **315**, 1687-1691. (10.1126/science.1139393) 17332376

[85] Borsa M et al. 2019 Modulation of asymmetric cell division as a mechanism to boost CD8+ T cell memory. Sci. Immunol. **4**, eaav1730. (10.1126/sciimmunol.aav1730) 30979796

[86] Zhou D, Luo Y, Dingli D, Traulsen A. 2019 The invasion of de-differentiating cancer cells into hierarchical tissues. PLoS Comput. Biol. **15**, e1007167. (10.1371/journal.pcbi.1007167) 31260442 PMC6625723

[87] Ciofani M, Zúñiga-Pflücker JC. 2010 Determining *γδ* versus *αβ* T cell development. Nat. Rev. Immunol. **10**, 657-663. (10.1038/nri2820) 20725107

[88] Crompton T, Outram SV, Hager-Theodorides AL. 2007 Sonic hedgehog signalling in T-cell development and activation. Nat. Rev. Immunol. **7**, 726-735. (10.1038/nri2151) 17690714

[89] Seddon B, Yates AJ. 2018 The natural history of naive T cells from birth to maturity. Immunol. Rev. **285**, 218-232. (10.1111/imr.12694) 30129206

[90] Lythe G, Callard RE, Hoare RL, Molina-París C. 2016 How many TCR clonotypes does a body maintain? J. Theor. Biol. **389**, 214-224. (10.1016/j.jtbi.2015.10.016) 26546971 PMC4678146

[91] Gonçalves P, Ferrarini M, Molina-Paris C, Lythe G, Vasseur F, Lim A, Rocha B, Azogui O. 2017 The selection for full diversity: a new mechanism shapes the naïve CD8+ T cell repertoire. Mol. Immunol. **85**, 66-80. 28212502 10.1016/j.molimm.2017.01.026

[92] Lythe G, Molina-París C. 2018 Some deterministic and stochastic mathematical models of naïve T-cell homeostasis. Immunol. Rev. **285**, 206-217. (10.1111/imr.12696) 30129198

[93] Desponds J, Mora T, Walczak AM. 2016 Fluctuating fitness shapes the clone-size distribution of immune repertoires. Proc. Natl Acad. Sci. USA **113**, 274-279. (10.1073/pnas.1512977112) 26711994 PMC4720353

[94] de Greef PC, Oakes T, Gerritsen B, Ismail M, Heather JM, Hermsen R, Chain B, de Boer RJ. 2020 The naive T-cell receptor repertoire has an extremely broad distribution of clone sizes. Elife **9**, e49900. (10.7554/eLife.49900) 32187010 PMC7080410

[95] Mukhopadhyay M. 2022 Reporting T cell proliferation. Nat. Methods **19**, 521-521. (10.1038/s41592-022-01505-3) 35545710

[96] Feliciangeli F, Dreiwi H, López-García M, Castro Ponce M, Molina-París C, Lythe G. 2022 Why are cell populations maintained via multiple compartments? Zenodo. (10.5281/zenodo.7181108) PMC9653237 36349449

